# Properties, Origin, and Consistency of Truncated Proteoforms Across Top-Down Proteomic Studies

**DOI:** 10.1016/j.mcpro.2025.101465

**Published:** 2025-11-12

**Authors:** Philipp T. Kaulich, James M. Fulcher, Andreas Tholey

**Affiliations:** 1 Systematic Proteome Research & Bioanalytics, Institute for Experimental Medicine, Christian-Albrechts-Universität zu Kiel, Kiel, Germany; 2 Environmental Molecular Sciences Laboratory, Pacific Northwest National Laboratory, Richland, Washington, USA

**Keywords:** top-down proteomics, truncation, proteolysis, experimental artifacts, meta-analysis

## Abstract

Protein truncation is a common modification that can alter protein localization, interaction, activity, and function. Top-down proteomics targets the identification of all molecular forms in which a protein can exist (termed “proteoforms”) and is thus well-suited for termini analysis. To examine the properties, origin, and consistency of truncated proteoforms, we performed a meta-analysis of 50 top-down proteomics datasets published over the past decade, covering 140,000 proteoforms derived from 14,500 proteins across various species. On average across all datasets, approximately 71% of proteoforms were truncated, with the vast majority not yet being documented in protein databases. Our analysis was able to distinguish between artificial truncations (*e.g.*, sample preparation effects on labile peptide bonds) and endogenous truncations, enabling the identification of novel signal peptides and truncations between structured domains. This study highlights the importance of a common yet understudied mechanism for generating protein diversity and provides a valuable resource for future studies, targeting truncated proteoform functions or aiming to reduce artefacts in proteomics sample preparation.

## Highlights

• Meta-analysis examining >140,000 proteoforms from 50 top-down proteomics datasets.
• Most reported proteoforms are truncated and not documented in protein databases.
• Biological and methodological factors influence truncated proteoform identification.
• Sample preparation can introduce artificially truncated proteoforms.
• Consistent identification across independent datasets hints at biological relevance.

## Graphical Abstract

**Figure:**
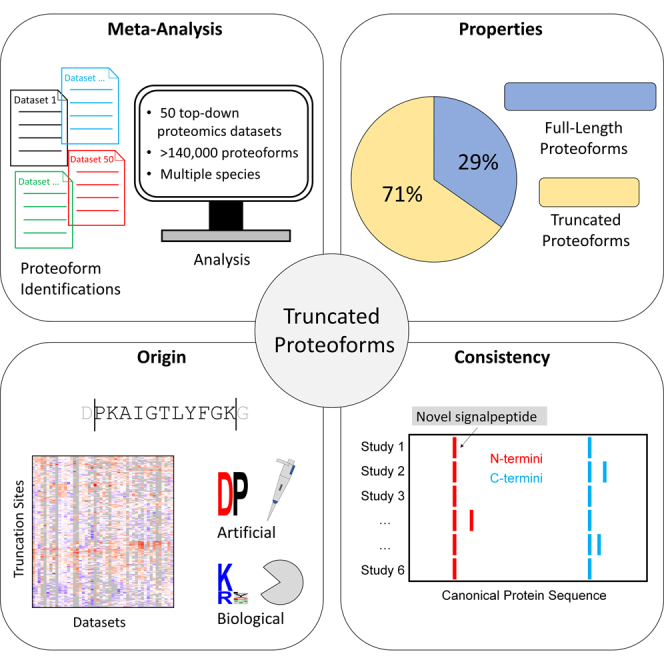

## In Brief

We performed a meta-analysis of 50 top-down proteomics datasets published over the past decade, covering 140,000 proteoforms derived from 14,500 proteins across various species to examine the properties, origin, and consistency of truncated proteoforms. This study highlights the importance of a common yet understudied mechanism for generating protein diversity and provides a valuable resource for future studies, targeting truncated proteoform functions or aiming to reduce artefacts in proteomics sample preparation.

Proteoforms are the different molecular forms in which proteins exist, arising from transcriptional, cotranslational, and posttranslational modifications (PTMs) (1). A common modification is truncation (2), which can occur at single or multiple sites in a protein (3). Biologically, these truncations may result from transcriptional or translational processes, such as alternative splicing, alternative translation initiation, and premature termination (4, 5, 6), and at the posttranslational level, such as the hydrolysis of amide bonds by proteases (7).

Truncation can significantly impact the physicochemical properties and interactions between proteins and, therefore, the biological activity, subcellular localization, stability, and biological function (8, 9, 10, 11). Not surprisingly, proteolytic processing plays a crucial role in many biological processes. For example, a common event is membrane protein ectodomain shedding (12), where the proteolytic processing of a membrane-binding domain results in a change in proteoform localization. Furthermore, truncated proteoforms are known to play critical roles in cellular processes such as mitochondrial complex activity (13) and cell cycle progression (14), as well as in human diseases, including cancer and Alzheimer's disease (15, 16, 17).

Thus, understanding truncated proteoforms is crucial for deciphering the full complexity of the proteome and the underlying molecular processes within cells. To date, the majority of proteomics analyses are based on bottom-up proteomics (BUP), where proteoforms are enzymatically digested into peptides and analyzed typically by LC-MS, after which peptide-level information is used to infer protein groups (18). While BUP is sensitive, it fails to deliver complete proteoform-level information (19), and, in particular, details about proteoform termini are lost (20). To circumvent the latter, terminomics approaches have been developed to identify proteoform termini (21, 22, 23, 24, 25, 26). These techniques typically involve labeling the termini and depleting internal peptides or enriching terminal peptides after digestion, significantly increasing proteome termini coverage.

However, an inherent drawback of all BUP-based (terminomics) methods is the loss of information regarding the connection between the proteoform N and C terminus. In contrast, top-down proteomics (TDP) targets intact proteoforms, preserving the complete proteoform information, including both termini (27). Although TDP is well-suited for analyzing proteoform termini (7), only a limited number of studies have focused explicitly on truncated proteoforms (16, 28, 29, 30). Recent examples include tissue-specific truncation sites identified through TDP (28), characterization of proteases responsible for the truncations of extracellular vesicle proteoforms (29), and the differential association of amyloid beta truncations with amyloid plaques and cerebral amyloid angiopathy in Alzheimer's disease (16).

Although TDP can derive simultaneous information on both termini for a given proteoform, several caveats have been identified in this approach, including limited coverage of the proteome, low overlap with termini identified by BUP, and challenging data evaluation of TDP data when labeling strategies are applied (31). Moreover, besides the biological origin, truncated proteoforms can also be formed artificially during sample preparation, proteoform separation, or mass spectrometry (MS) analysis (32, 33, 34). For example, it has been shown that different cell lysis conditions influence the occurrence of truncated proteoforms, for example, due to active proteases (35). Moreover, elevated temperatures and acidic conditions have been reported to introduce artificial proteoforms (32, 33). Therefore, distinguishing between biological and artificial protein truncations in proteomics studies remains a major challenge.

In the present study, we introduced the pooled analysis of protein truncations (PAPT) to systematically investigate the characteristics of truncated proteoform termini reported in recent TDP studies (Supplemental Fig. S1). For this meta-analysis, we analyzed more than 140,000 proteoforms reported by 45 different TDP studies published over the past decade (16, 28, 29, 31, 32), (36, 37, 38, 39, 40, 41, 42, 43, 44, 45), (46, 47, 48, 49, 50, 51, 52, 53, 54, 55, 56, 57, 58, 59, 60, 61, 62, 63, 64, 65), (66, 67, 68, 69, 70, 71, 72, 73, 74, 75). Specifically, we analyzed the proteoforms in terms of their termini, truncation sites, and consistency. Some datasets exhibit similar truncation site patterns, which could be attributed to artificially introduced modifications during sample preparation. Numerous noncanonical termini of certain proteins were consistently identified in various independent TDP studies, enabling the correction of previously described signal peptides or the identification of novel ones. Furthermore, truncations between protein domains were a commonly observed phenomenon. The results of the PAPT provide valuable insights into the characterization and interpretation of truncated proteoforms. Additionally, this meta-analysis can serve as a valuable resource for identifying relevant proteoforms of specific proteins, providing starting points for further biological investigations into their functional roles and regulatory mechanisms.

## Experimental Procedures

### Experimental Design and Statistical Rationale

TDP studies published between 2016 and 2025 providing easily accessible proteoform identifications, including at least the proteoform sequence and the corresponding protein accession, were selected. The studies covered a variety of laboratories, samples (organisms and sample type), sample preparation approaches (*i.e.*, cell lysis conditions, enrichment of suitable proteoforms for TDP approaches, and fractionation strategies), data acquisition strategies (mass analyzers and mass spectrometric settings), and proteoform identification pipelines (proteoform database, deconvolution, search algorithms, and database search settings) (Table 1).

**Table 1:** Overview of the datasets utilized in this meta-analysis for the investigation of truncated proteoforms

Dataset	DOI	Organism	Mass analyzer	Search engine
DS-01	10.1126/science.aaz5284	Human	Orbitrap	TDPortal
DS-02	10.1021/acs.jproteome.0c00403	Human	Orbitrap	TDPortal
DS-03	10.1126/sciadv.abq6348	Human	Orbitrap	TopPic
DS-04	10.1007/s13361-019-02315-2	Human	Orbitrap	TopPic
DS-05	10.1021/acs.analchem.2c02777	Human	Orbitrap	ProSightPD
DS-06	10.1021/acs.jproteome.0c00226	Human	Orbitrap	TopPic
DS-07	10.1021/acs.jproteome.6b00696	Human	FT-ICR	TDPortal
DS-08	10.1016/j.mcpro.2025.100983	Human	Orbitrap	TopPic
DS-09	10.1021/acs.analchem.0c02533	Human	Q-TOF	TopPic
DS-10	10.1021/acs.jproteome.3c00488	Human	Orbitrap	ProSightPD
DS-11	10.1021/acs.analchem.2c03045	Human	Orbitrap	TopPic
DS-12	10.1002/pmic.202200542	Human	Orbitrap	ProSightPD
DS-13	10.1038/s41592-024-02481-6	Human	Orbitrap	ProSightPD
DS-14	10.1021/acs.analchem.1c05123	Human	Orbitrap	ProSightPD
DS-15	10.1021/acs.jproteome.5b00997	Human	Orbitrap	ProSightPC
DS-16	10.1021/acs.jproteome.2c00034	Human	Orbitrap	TDPortal
DS-17	10.1021/acs.jproteome.6b00698	Human	Orbitrap	TDPortal
DS-18	10.1021/acs.analchem.0c00467	Human	Orbitrap	TopPic
DS-19	10.1021/acs.jproteome.1c00049	Human	Orbitrap	TopPic
DS-20	see DS-08	*Mus musculus*	see DS-08	
DS-21	10.1021/acs.analchem.2c05401	*M. musculus*	Orbitrap	TopPic
DS-22	10.1186/s12014-024-09509-1	*M. musculus*	Orbitrap	TopPic
DS-23	10.1210/endocr/bqad160	*M. musculus*	Orbitrap	TopPic
DS-24	10.1021/acs.analchem.3c02346	*M. musculus*	Orbitrap	TopPic
DS-25	10.1021/acs.jproteome.9b00487	*M. musculus*	Orbitrap	ProSightPD
DS-26	10.1016/j.mcpro.2022.100491	*Rattus norvegicus*	Orbitrap	TDPortal
DS-27	10.1021/acs.jproteome.2c00277	*Caenorhabditis elegans*	Orbitrap	ProSightPD
DS-28	10.1002/anie.202301969	*C. elegans*	Orbitrap	ProSightPD
DS-29	10.1039/D1MO00335F	Zebrafish	Orbitrap	TopPic
DS-30	10.1007/s13361-019-02167-w	Zebrafish	Orbitrap	TopPic
DS-31	10.1021/acs.analchem.9b05578	Zebrafish	Orbitrap	TopPic
DS-32	10.1002/pmic.202100377	*Arabidopsis thaliana*	Orbitrap	TopPic
DS-33	10.3389/frans.2022.1012707	*Bradyrhizobium japonicum/Soybean*	Orbitrap	TopPic
DS-34	10.1021/acs.jproteome.3c00872	Yeast	Orbitrap	TopPic
DS-35	10.1038/s41592-019-0391-1	*Sus scrofa*	Q-TOF	TopPic
DS-36	10.1021/acs.analchem.8b00693	*Escherichia coli*	Orbitrap	TopPic
DS-37	10.1021/acs.analchem.3c01534	*E. coli*	Orbitrap	ProSightPD
DS-38	10.1016/j.aca.2022.340037	*E. coli*	Orbitrap	TopPic
DS-39	see DS-06	*E. coli*	see DS-06	
DS-40	see DS-30	*E. coli*	see DS-30	
DS-41	10.1021/acs.analchem.0c03395	*E. coli*	Orbitrap	TopPic
DS-42	10.1021/acs.jproteome.1c00460	*E. coli*	Orbitrap	ProSightPD
DS-43	see DS-42	*E. coli*	see DS-42	
DS-44	see DS-31	*E. coli*	see DS-31	
DS-45	10.1021/acs.analchem.4c01119	*E. coli*	Orbitrap	TopPic
DS-46	10.1021/acs.jproteome.0c00303	*E. coli*	FT-ICR	TDPortal
DS-47	10.1128/spectrum.02528-23	*Blautia producta*	Orbitrap	ProSightPD
DS-48	10.1038/s41598-023-29857-6	*Corynebacterium glutamicum*	Orbitrap	TopPic
DS-49	10.1021/acs.jproteome.0c00351	Multiple Bacteria	Orbitrap	ProSightPD
DS-50	10.1016/j.jprot.2020.103988	*Methanosarcina mazei*	Orbitrap	ProSightPD

DOI, digital object identifier; FT-ICR, Fourier-transform ion cyclotron resonance; Q-TOF, quadrupole-time of flight.

Several TDP studies provided multiple proteoform identification lists, such as replicate measurements, different sample preparations, various acquisition strategies, or distinct biological samples. For the general analysis of the truncations, one representative dataset was selected from each study, typically the dataset with the highest number of identifications. Only in cases where the datasets within a study differed significantly from each other were several datasets of a single study selected, for example, if different organisms were analyzed or strongly complementary proteoform identifications were obtained. The nomenclature of the dataset throughout the text is dataset-number, where NN is a consecutive number. Table 1 provides general information about the datasets, including the digital object identifier of the publication, the organism analyzed, and the mass analyzer and database search utilized.

To investigate specific research questions, such as the intra-study reproducibility and the influence of different biological conditions or sample preparation steps on truncated proteoform identification, appropriate datasets from individual TDP studies were used. These datasets were designated as dataset-number-x, where x is a letter assigned in ascending alphabetical order (Supplemental Table S1).

### Data Analysis

#### Preparation of the Datasets

The proteoform identifications of the analyzed studies were extracted, for example, from the Supplemental Tables or downloaded from data repositories such as PRIDE (76). The minimum information required was the proteoform sequence and the associated protein accession. This information and, if available, the ProForma standard notation (77), the number of proteoform spectrum matches (PrSMs), and the start and end positions of the proteoform with respect to the canonical sequence were extracted from the proteoform identification lists for the meta-analysis (Supplemental Table S2).

Information about previously described truncations was obtained from the UniProt Knowledgebase by downloading the data on reported protein accessions in XML format (release 2024_01) (78). Only proteoforms with active protein accessions were considered for further analysis. In rare cases, datasets included proteoforms that were not associated with any protein accession, or the reported protein accession was no longer present in the UniProt database (79). This can occur because UniProt regularly removes entries, such as when a sequence prediction is deleted from Ensembl or RefSeq, when an accession is merged into a new one, or if the entry has been divided into separate entries. If no valid proteoform sequence or protein accession could be assigned, the corresponding proteoform identifications were removed prior to further analysis.

#### Determination of Proteoform Truncation State

To investigate if a proteoform is truncated, the start and end positions relative to the canonical sequence were analyzed by matching the reported proteoform sequence to the canonical protein sequence deposited in UniProt. The proteoforms were assigned to full-length proteoforms if the sequence covered both the canonical N terminus (or the cleaved start-methionine) and the C terminus. In contrast, exclusive N terminally truncated proteoforms retained only the canonical C terminus, and vice versa, exclusive C terminally truncated proteoforms retained the canonical N terminus. Proteoforms that did not cover the canonical N and C termini were classified as N and C terminally truncated proteoforms. Note that the excision of the start-methionine was not considered as truncation.

#### Determination of the Proteoform Truncation Site and Normalization

The truncation sites were determined by mapping the reported truncated proteoform sequence to the canonical sequence deposited in the UniProt database. The amino acids N and C terminal to the truncations were denoted as X and X' (adapted from the nomenclature of Schlechter and Berger) (80), respectively. Moreover, to investigate differences between the two termini, the truncation sites of the truncated N terminus were denoted as X_N_ and X_N_', and of the truncated C terminus as X_C_ and X_C_'.

**Fig. 1:**
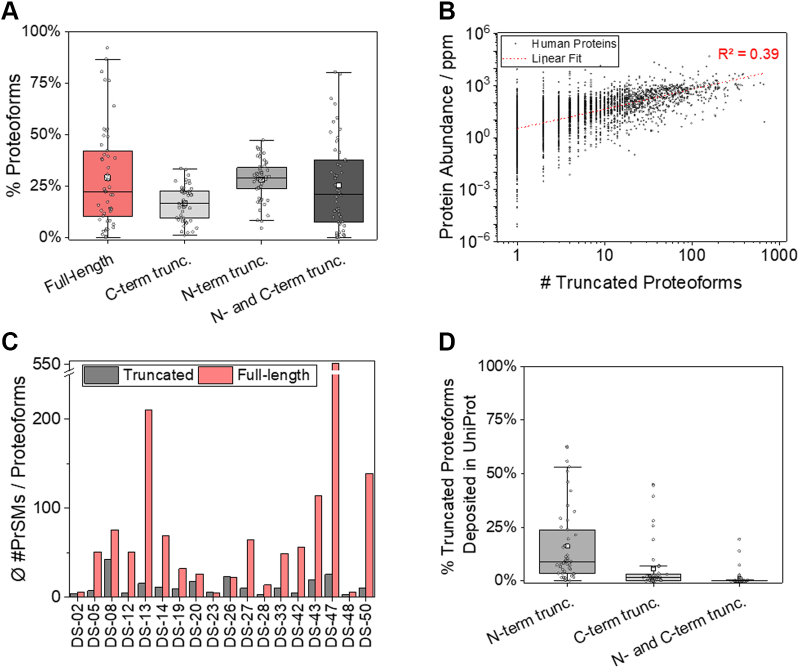
T**runcated proteoforms identified in current TDP studies**. *A*, distribution of full-length and truncated proteoforms reported in the datasets (n = 50). *B*, correlation of protein abundance (according to the PaxDb) and the number of truncated proteoforms across human datasets. *C*, average number of proteoform spectrum matches (PrSMs) that have been assigned to full-length and truncated proteoforms (only datasets that provided PrSM information are included). For each dataset, the total number of PrSMs assigned to truncated and full-length proteoforms was divided by the number of identified truncated and full-length proteoforms, respectively. *D*, percentage of truncated proteoforms reported by the various studies documented in the UniProt database. Boxplots: 25 to 75%; error bars: 1.5 interquartile range; *black line*: median; *white rectangle*: mean; *gray dots*: data points, n = 50. TDP, top-down proteomics.

**Fig. 2:**
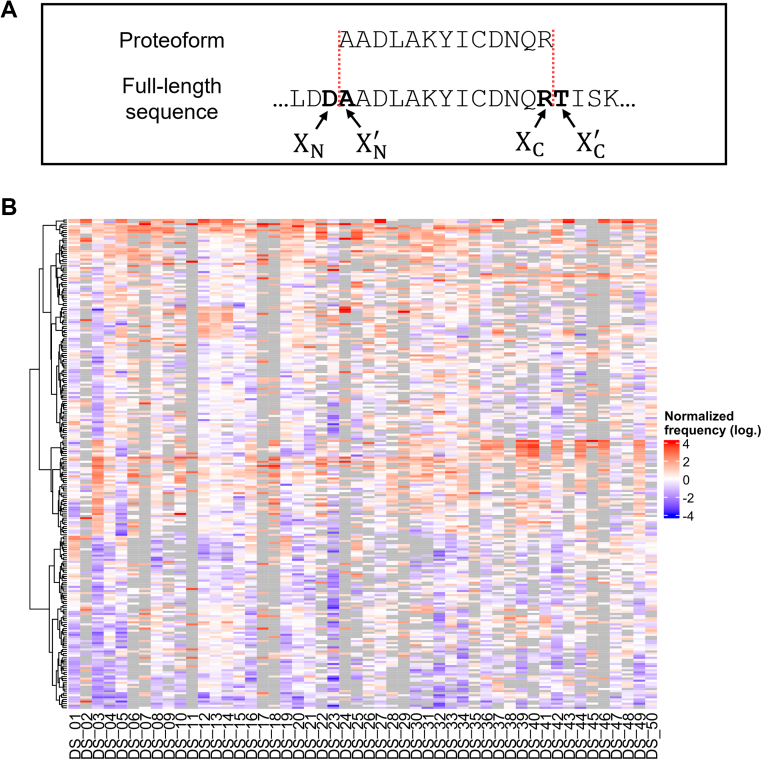
**Truncation sites of proteoforms across all analyzed TDP studies**. *A*, truncated proteoforms were matched to the full-length sequence, and the amino acids N and C terminal of the truncation site(s) were denoted as X and X ′, respectively, and can be further divided into the truncation site at the proteoform N terminus (X_N_ and X_N_') and C terminus (X_C_ and X_C_'). *B*, heat map of truncation sites (X_N_|X_N_' and X_C_|X_C_', *y-*axis) with fewer than 40% missing values across the 50 datasets (*x*-axis), representing their frequencies normalized on the number of corresponding peptide bonds in each dataset. A value of zero (*white*) indicates that the truncation occurs with the same relative frequency as the peptide bond in the proteoforms. A positive value (*red*) indicates an overrepresented truncation site, meaning that the peptide bond is cleaved more frequently than expected based on its occurrence, while a negative value (*blue*) indicates an underrepresented truncation site. Missing values are represented with *gray*. TDP, top-down proteomics.

Normalization of the number of truncation sites was performed to facilitate a comparison of multiple datasets (Supplemental Table S3). To this end, the number of truncation events between specific amino acid residues was divided by the number of corresponding peptide bonds identified in the dataset. Calculating the logarithm of this value is a measure of the normalized frequency of a given truncation site.

#### Clustering and Visualization of Proteoform Truncation Sites and Datasets

Normalized frequencies of truncated N termini (X_N_|X_N_') and of truncated C termini (X_C_|X_C_') from Supplemental Table S3 were imported into the R environment (v.4.5.0) using R Studio (v.2025.05.1+513). Datasets and observations (*i.e.*, termini truncations) were filtered to those with fewer than 40% missing values to allow for hierarchical clustering. Hierarchical clustering was performed with Euclidean distances through hclust( ) with Ward's criterion (“ward.D2”), and the generated heat maps were visualized using the R package ComplexHeatmap (v.2.25.2) (81). Truncation clusters were defined by cutting the dendrogram at k = 15. The dendrogram was produced using the R package dendextend (v.1.19.0) (82) and the cluster and enrichment motif table was created using the R package gt (v.1.0.0.9) in combination with motif enrichment using default settings in WebLogo 3 (83).

**Fig. 3:**
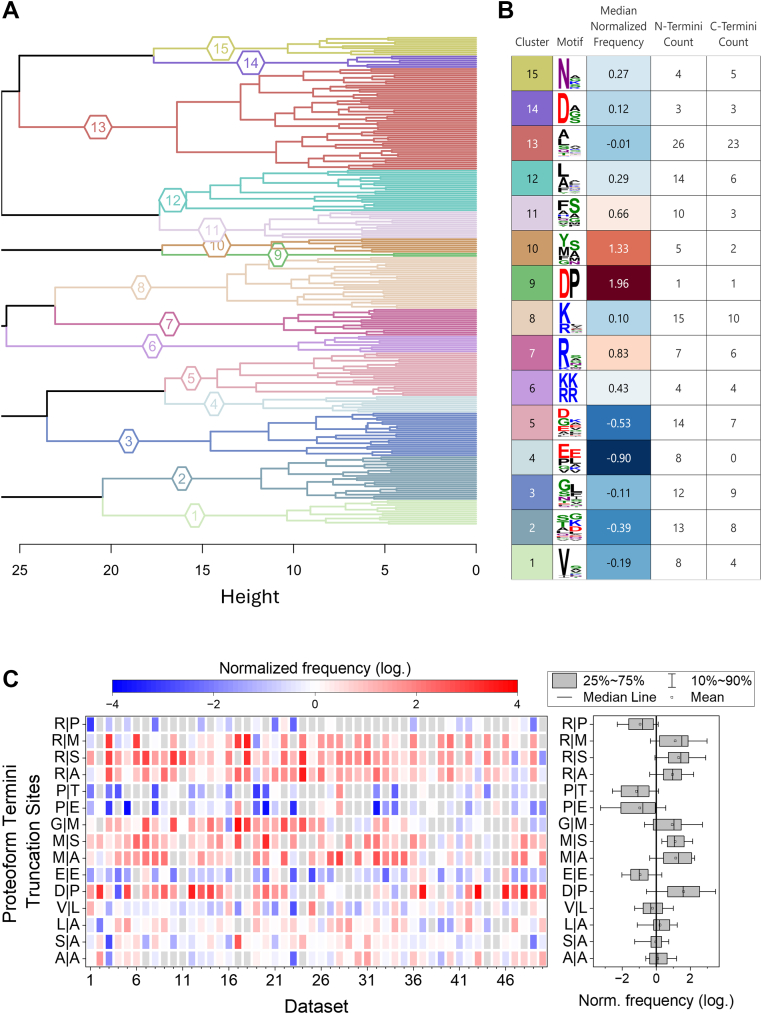
**Relationship between truncation events and overrepresented or underrepresented truncation sites across all datasets**. *A*, dendrogram of hierarchically clustered (k = 15) truncation sites based on their normalized frequencies. Truncation sites were filtered to those having less than 40% missing values. Colors indicate clusters present in the dendrogram. *B,* table of clusters presenting the cluster assignment from (*A*), corresponding amino acid motif enrichment (performed with WebLogo 3) (83), median normalized frequency of occurrence across the clustered truncation sites, and the count of truncation sites that occur at the N terminus or C terminus of the proteoform. *C*, the heat map highlights selected truncation sites that are overrepresented or underrepresented in most datasets or have inconsistent normalized frequencies across datasets. For the normalized frequencies, *red* indicates overrepresented truncation sites, while *blue* indicates underrepresented truncation sites.

### LC-MS/MS Analysis

LC-MS/MS measurements were performed to experimentally validate the influence of in-source ion activation on the artificial generation of truncated proteoforms. Proteoform separation was performed with a Dionex U3000 UHPLC system (Thermo Fisher Scientific) equipped with a reversed-phase Accucore C4 column (50 cm × 75 μm, 2.6 μm, 150 Å, Thermo Fisher Scientific). Eluent A was 0.05% (v/v) formic acid, and eluent B was 80% (v/v) acetonitrile, 0.04% (v/v) formic acid. The proteoforms were separated using a 120-min gradient (300 nl/min flow rate, 45 °C) from 15% B to 60% B: 0 to 5 min, 4% B; 5 to 7 min, 4 to 15% B; 7 to 127 min, 15 to 60% B; 127 to 129 min, 60 to 90% B; 129 to 140 min, 90% B; 140 to 140.1 min, 90 to 4% B; 140.1 to 150 min, 4% B. The liquid chromatography (LC) was coupled online to a Fusion Lumos Tribrid mass spectrometer (Thermo Fisher Scientific) equipped with a high-field asymmetric waveform ion mobility spectrometry (FAIMS) Pro interface, and an optimized multi-compensation voltage (CV) method was utilized (47). In brief, 4 CVs were used (−60, −50, −40, and −25 V) with optimized resolutions and maximum injection times. Within 3 s, the most intense precursors (charge states: 4–50, including undetermined charge states; dynamic exclusion (n = 2, ±1.5 *m/z*, 60 s) was enabled) were fragmented using collision-induced dissociation (CID) with a normalized collision energy of 25%. Approximately 500 ng of *Escherichia coli* proteome enriched for proteoforms smaller than approximately 20 kDa using solid-phase extraction (32) was injected, and the in-source ion activation energy (“in source fragmentation energy”) was varied between 0 and 80 V. All raw data acquired in this study have been uploaded to the ProteomeXchange Consortium (76) via the PRIDE partner repository with the dataset identifier PXD066719.

### Deconvolution and Database Search for Proteoform Identification

Database searches for the analysis of the influence of in-source ion activation and reanalysis of datasets (DS-16: MSV000088565, MassIVE; DS-14: PXD029792, PRIDE, only FAIMS data) were performed using ProSightPD (v.4.2, Proteinaceous) (84) within ProteomeDiscoverer (v.3.0.0.757, Thermo Fisher Scientific). The raw files were deconvolved using Xtract with the ProSightPD High/High cRAWler node, employing default settings. If necessary, a precursor spectral shift was applied to the MS1 and MS2 levels. If not otherwise stated, the deconvolved masses were searched against a proteoform database containing all known modifications (downloaded as an XML or FASTA file from UniProt (78); human datasets: taxon ID 9606, release 2023_01; *E. coli* datasets: taxon ID 83333, release 2022_01) using the annotated and subsequence search. Variable methionine excision and acetylation or formylation at the canonical N terminus were allowed. If not stated otherwise, the precursor and fragment tolerance were set to 10 ppm, and the false discovery cutoff was set to 1% (85).

Furthermore, to investigate the influence of the database search engine, proteoform identification (DS-14: PXD029792, PRIDE, only FAIMS data) was performed with the standalone software suite TopPIC (v.1.7.3) (86) using the default workflow. In brief, the raw data were converted to the mzML file format using msConvert (87) and deconvolved with TopFD. The deconvolved spectra were searched against a human protein database (downloaded as a FASTA file from UniProt, taxon ID 9606, release 2023_01). Variable methionine excision and acetylation at the canonical N terminus were allowed. The precursor tolerance was set to ±500 Da, the fragment mass tolerance to 10 ppm, and the false discovery cutoff was set to 1%.

### Various

All data analyses were performed using Python (v.3.11.10) or R (v.4.5.0). Signal peptide prediction was performed based on the proteoform sequence using TargetP (v2.0) (88). The protein abundance values were extracted from the Protein Abundances Across Organisms Database using the *Homo sapiens* whole organism (integrated) dataset (89). For analyzing mass features, the raw files were converted to the mzML file format using msConvert (87) and deconvolved using FLASHDeconv (90) with default settings, except for the minimal mass (3000 Da) and charge range (3–50).

## Results

### Overview of the Datasets

For the PAPT, we selected 50 datasets from 45 in-depth TDP studies published over the past decade that provided easily accessible proteoform identification lists (Table 1, Supplemental Table S1). The number of datasets exceeds the number of studies because some studies included multiple datasets, for example, from different organisms or experimental conditions. We used the proteoforms reported in the original studies for our meta-analysis; that is, no additional database searches were performed.

The datasets represented various species, with the highest numbers being *H. sapiens* (n = 19) and *E. coli* (n = 11) datasets, and were acquired by different laboratories using numerous sample preparation approaches, three different classes of mass analyzers (Orbitrap, Fourier-transform ion cyclotron resonance, and quadrupole-time of flight), and three database search pipelines (TopPIC, ProSightPD, and TDPortal). From 18 (36%) of the datasets, the number of PrSMs could be extracted for a semiquantitative analysis.

In total, more than 140,000 proteoforms derived from 14,500 proteins were analyzed (Supplemental Table S2), with a median of 1485 proteoforms associated with 307 proteins per dataset (Supplemental Fig. S2). The reported proteoform sequences were matched to their canonical sequence in the UniProt database (78), and the truncation state was determined. N-terminal start-methionine excision was not considered a truncation. Note that, without additional data integration, confidently distinguishing whether a truncation is the product of a transcriptional, translational, posttranslational, or artifactual event is challenging based solely on the identification of proteoforms by MS. Therefore, the use of the term “truncation” is intended to summarize all events that can lead to truncated proteoforms.

### Properties of Truncated Proteoforms

The number of truncated proteoforms reported in a given study was positively correlated with the overall number of identified proteoforms (Supplemental Fig. S2). Across all datasets, approximately 29 ± 3% (average ± SEM, n = 50) of the reported proteoforms were full-length (Fig. 1*A*). Among the truncated proteoforms, these could be divided into groups that were exclusively N terminally truncated (28 ± 1%), N and C terminally (26 ± 3%), and exclusively C terminally truncated (17 ± 1%) (Supplemental Fig. S3*A*). At the protein level, 26 ± 3% of the proteins were identified exclusively in their full-length form, while 60 ± 3% of the proteins were identified solely with truncated proteoforms. Only 14 ± 1% of the proteins were identified both with truncated and full-length proteoforms (Supplemental Fig. S3*B*).

Theoretically, a longer encoded protein sequence can generate a higher number of truncated proteoforms; however, the number of identified truncated proteoforms of a given protein was not correlated with its encoded size (Supplemental Fig. S4*A*). In contrast, a positive correlation (R^2^ = 0.39) was observed between the abundance of a protein (according to the Protein Abundances Across Organisms Database) (89) and the number of truncated proteoforms identified (Fig. 1*B*). The highest number of truncated proteoforms (n = 600) was reported for cytoplasmatic actin 1 (P60709) in DS-01, which is one of the top 30 most abundant proteins in human cells (89). Moreover, the smaller the proteoform sequence, the higher the proportion of truncated ones (Supplemental Fig. S4*B*). For example, within the length range of 125 to 150 amino acids, 73% of the proteoforms were full-length ones, whereas only 3% were N and C terminally truncated. Conversely, within the length range of 25 to 50 amino acids, only 2% of the proteoforms were full-length ones, and 49% were N and C terminally truncated.

Studies that reported very high or low proportions of truncated proteoforms compared to the average utilized, in some cases, specialized experimental designs. For example, DS-47 and DS-50 were designed to analyze short ORF-encoded peptides, thereby enriching the subfraction of small proteins and potentially contributing to the identification of a higher percentage of truncated proteoforms (Supplemental Figs. S3*A* and S4*B*). On the other hand, DS-17 focused on identifying large proteoforms by applying a low-resolution MS1 acquisition scheme, which contributed to an increased percentage of full-length proteoforms. Moreover, studies utilizing Fourier-transform ion cyclotron resonance (DS-07, DS-46) or TOF (DS-09, DS-35) mass analyzers tended to identify a lower percentage of truncated proteoforms than Orbitrap instruments.

We estimated the relative abundance of truncated compared to full-length proteoforms by using the number of assigned spectra (PrSMs) as a rough indicator for their abundance (91). For almost all 18 datasets that reported this parameter, the number of assigned PrSMs was much higher for full-length than truncated proteoforms (Fig. 1*C*). Randomly selected examples of proteins identified with both full-length and truncated proteoforms are shown in Supplemental Figure S5: While many truncated proteoforms were identified, the majority of PrSMs were assigned to the full-length proteoform. These findings indicate that full-length proteoforms are generally more abundant than individual truncated proteoforms derived from the same protein. Furthermore, this is in agreement with the observation that studies with reduced sensitivity (*i.e.*, reporting a limited number of proteoforms) primarily identify full-length (*i.e.*, highly abundant) and less truncated (*i.e.*, less abundant) proteoforms (Supplemental Figs. S2 and S3). For example, in a single-cell TDP study (DS-11), of the 83 identified proteoforms, 67 were full-length ones.

We analyzed the positions of the proteoforms' N and C termini in relation to their corresponding canonical sequences (Supplemental Fig. S6). For N-terminal truncated proteoforms, the majority were found to start at position 1 (*i.e.*, the canonical start-methionine) and position 2 (*i.e.*, the canonical terminus after start-methionine excision). Significantly fewer proteoforms started at position 3, which may be explained by additional aminopeptidase activity. Notably, the majority of proteoforms whose N terminus starts at position 3 originate from DS-01, which analyzed human blood, a sample type known to exhibit exopeptidase activity (92). These observations indicate that aminopeptidase activity is not a substantial general (but not a negligible) source of truncated proteoforms in current TDP studies.

Additionally, a considerable number of proteoforms exhibited N-terminal start positions between residues 19 and 30. This pattern was even more pronounced when the distribution of truncation site positions was analyzed within individual studies. Manual inspection of the human datasets DS-01, DS-08, and DS-13 revealed that many of these truncation sites align with the cleavage of signal or transit peptides deposited in the UniProt database (Supplemental Fig. S6, *A* and *B*).

For the C terminus, the majority of proteoforms were reported at the canonical C terminus (position 0, Supplemental Fig. S6, *C* and *D*). Only a limited number of proteoform C termini at position −1 (*i.e.*, excision of the canonical C-terminal amino acid) were reported, originating, for example, due to carboxypeptidase activity (mainly from DS-01 and DS-13). No other notable preferred position in the primary structure for the positions of the proteoform C termini was observed.

### Proteoform Truncations Annotated in UniProt

Only 16 ± 2% (average ± SEM, n = 50), 6 ± 2%, and 1.1 ± 0.5% of the N terminally, C terminally, and N/C terminally truncated proteoforms reported in the studies are described in the UniProt database (Fig. 1*D*, Supplemental Fig. S7*A*), highlighting the limited knowledge about truncated proteoforms. Moreover, the abundance (based on the number of PrSMs) of known truncated proteoforms was substantially higher than unknown ones (Supplemental Fig. S7*B*).

The outliers, that is, those studies that reported substantially higher percentages of known truncation events, could be explained by the specific sample type analyzed, such as certain cell organelles (*e.g.*, chloroplasts, DS-32) or tissues (*e.g.*, pancreatic islet cells, DS-23) (Supplemental Fig. S7*A*). Many chloroplast proteins possess transit peptides that are cleaved after the protein transport, resulting in N terminally truncated proteoforms. Pancreatic islet cells are known to contain numerous peptide hormones that are proteolytically processed to remove propeptides, signal peptides, and, in many cases, internal regions. In both cases, many highly abundant and well-known (and, thus, already archived in UniProt) truncated proteoforms were expectedly identified. Furthermore, studies that reported a limited number of truncated proteoforms identified a higher percentage of known truncation events. For example, in a single-cell TDP study (DS-11), 56% (n = 49) and 45% (n = 9) exclusively N- and C-terminal truncations, respectively, have already been deposited in UniProt. In contrast, in DS-01, which reported the highest number of proteoforms up to now, only 8% (n = 1106) and 1% (n = 169) of the N- or C-terminal truncations are described in UniProt.

### Effects of the Primary Structure on Truncations

Truncated proteoforms can arise either from biological processes or as artifacts introduced during sample preparation, data acquisition, or data processing. By leveraging the truncation sites of the proteoforms across all datasets, inferences can be made about the origin of proteoform truncations. Toward this end, the reported truncated proteoforms were matched with their canonical full-length sequences, and the amino acids adjacent to the truncation site(s) were determined. The amino acids N and C terminal to the truncation sites were denoted as X and X′, and can be further divided into the truncation sites at the proteoform N terminus (X_N_ and X_N_') and C terminus (X_C_ and X_C_') (Fig. 2*A*). Plotting the amino acids in X and X′ positions as two-dimensional histograms provides a global overview of the truncation sites in a given dataset (Supplemental Fig. S8). For example, DS-01, which analyzed human blood proteoforms, showed a slight bias toward proteoforms generated due to truncations C terminal to valine and alanine and N terminal to arginine, alanine, serine, and threonine residues (Supplemental Fig. S8*A*). In contrast, proteoforms from human cancer cells reported by DS-03 mainly originated from truncation C terminal to lysine and arginine residues (Supplemental Fig. S8*B*). Furthermore, by analyzing the amino acids in X_N_, X_N_', X_C_, and X_C_' position, conclusions about preferred truncation directions (93) and differences between the termini can be investigated (Supplemental Fig. S9, *A* and *B*).

To facilitate comparison across different datasets, we normalized the number of truncation sites (N-terminal X_N_|X_N_' and C-terminal X_C_|X_C_') based on the number of corresponding peptide bonds in each dataset (Supplemental Table S3). A positive value indicates that a peptide bond is truncated more frequently than expected based on its relative abundance (*i.e.*, overrepresented truncation), while a negative value indicates that it is truncated less frequently (*i.e.*, underrepresented truncation). If the truncations were evenly distributed across all peptide bonds, a value of zero would be expected. Observation of the corresponding heat map revealed substantial variability in these truncation events across the datasets (Fig. 2*B*).

To clarify the relationship between the different truncation events, we performed unsupervised hierarchical clustering (Fig. 3*A*) in combination with amino acid motif enrichment for identified clusters (Fig. 3*B*). Several truncation events exhibited clear trends in clustering, related to both their frequency of representation and amino acid motif. Specifically, these clusters could be grouped as such (i) C-terminal truncations to lysine and arginine residues in clusters 7 and 8 (K|X′ and R|X'); (ii) truncations between dibasic lysine and arginine residues in cluster 6 (K/R|K'/R'); (iii) truncations between aspartate-proline residues in cluster 9 (D|P′); (iv) C-terminal truncations to aspartate residues adjacent to small side-chain amino acids in cluster 14 (D|S'/G'/A'); (v) C-terminal truncations to asparagine residues in cluster 15 (N|G'/A'/S'/K'/R′); and (vi) C-terminal truncations to valine residues in cluster 1 (V|X'). As shown in Figure 3*B*, each cluster is presented with the number of N-terminal (X_N_|X_N_') and C-terminal (X_C_|X_C_') proteoform truncations. Most clusters showed no preference toward a particular terminus, except for cluster 4, which contained a number of truncations involving glutamic acid (and to a lesser extent, proline) exclusively at the N terminus of the proteoform.

The normalized frequencies of a few selected truncation events across all datasets are presented in Figure 3*C*. A number of clustered truncation events at sterically small, aliphatic amino acids, such as between alanine-alanine, serine-alanine, and leucine-alanine, and valine-leucine residues (clusters 12 and 13), showed no clear trend across all datasets. In some studies, they were overrepresented, while in others, they were underrepresented, resulting in a mean normalized frequency close to zero for these clusters. In contrast, truncation events that were overrepresented in nearly all datasets occurred between aspartate-proline (cluster 9), methionine-serine, and methionine-alanine residues (cluster 10), or C-terminal to arginine residues (clusters 6, 7, and 8) (Fig. 3*C*).

The aspartate-proline peptide bond is chemically labile, making it susceptible to high temperatures and acidic conditions (33, 94). Since many TDP workflows apply elevated temperatures (*e.g.*, during the reduction of disulfides) and acidic conditions (*e.g.*, during LC separation), the frequently observed truncation of the aspartate-proline bond (while also described as occurring *in vivo*) (95, 96) may be an artifact resulting from hydrolysis during sample preparation (32, 46).

The overrepresented truncation between methionine-serine and methionine-alanine hints toward the use of alternative translation initiation sites with subsequent methionine excision (9). Note that only truncated proteoforms were analyzed, that is, these truncation events are not derived from canonical initiator methionine residues. The hypothesis of alternative protein starting sites is further supported by the differences observed between the truncation sites of the proteoform N and C termini (Supplemental Fig. S9*C*), with the methionine-proline and methionine-serine peptide bonds being substantially more frequently truncated at the N terminus compared to the C terminus. In BUP-based terminomics studies, the N-terminal acetylation of novel N termini is commonly used to validate potential alternative translation initiation sites (9). However, here, the examination of modified truncated termini is currently hampered by limitations in database search algorithms for accurately identifying them, as well as the lack of comprehensive PTM annotations in many datasets.

Moreover, truncations C-terminal to arginine residues are significantly overrepresented in almost all datasets, with a notable exception being the underrepresented truncation between arginine and proline residues (Fig. 3*C*). This observation suggests potential proteolytic processing by specific proteases, of which several hydrolyze peptide bonds C terminal to arginine (97). On the other hand, peptide bonds involving proline residues are typically more resistant to proteolytic cleavage due to the conformational constraints imposed by the cyclic structure of proline (98). In line with this, truncations C terminal to proline are underrepresented in almost all datasets (Fig. 3*C*).

Note that, although multiple biases were observed for most datasets, there are studies in which certain biases are particularly pronounced. In the case of potentially artificially generated proteoforms, we hypothesize that sample preparation methods that favor peptide bond cleavages may be responsible for this phenomenon (*vide infra*). Additionally, certain sample types may exhibit unique characteristics that result in particularly pronounced truncation sites. In Supplemental Figure S10, some of these datasets, which show unique truncation site plots due to highly abundant proteins or the identification of many signal or transit peptides, are shown.

### Methodological Factors Influencing Truncated Proteoforms

Having investigated the relationships between the truncation sites and amino acid residues, we next systematically assessed methodological factors influencing the detection of truncated proteoforms across the TDP workflow. Note that the interpretability of the comparisons between different studies is severely limited due to significant variability among datasets, resulting from the wide range of sample types, sample preparation workflows, acquisition strategies, and data analysis pipelines used in varying combinations across studies, hampering the identification of single factors leading to specific truncations. Thus, we analyzed datasets obtained from individual studies that varied solely in a single biological or methodological parameter (Supplemental Table S1). Notably, the intra-study reproducibility of biological (*e.g.*, different biological replicates prepared with the same sample preparation) and technical (*e.g.*, multiple injections from the same sample) replicates regarding the truncated proteoforms was very high, that is, biological or technical variability did not substantially influence the truncations reported (Supplemental Fig. S11).

**Fig. 4:**
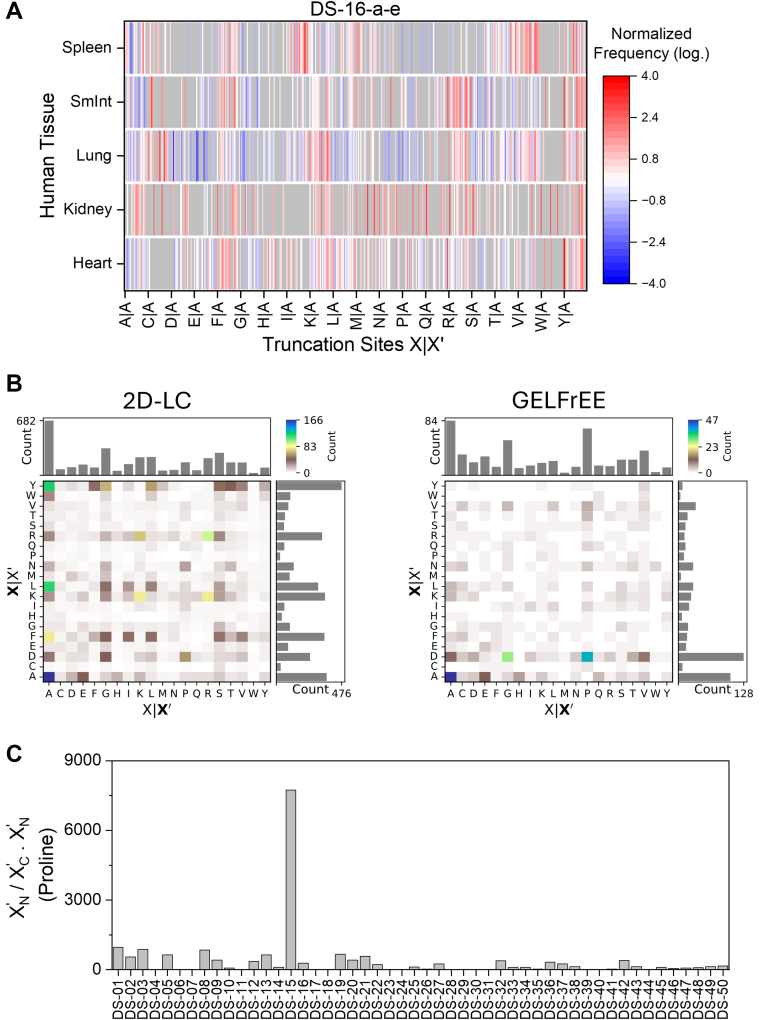
**Influence of the analytical workflow on the occurrence and identification of truncated proteoforms**. *A*, influence of the biological origin of the sample: Normalized frequency of truncation sites of truncated proteoforms identified in various human tissues in DS-16. *B*, influence of the sample preparation: The same *Escherichia coli* lysate was prepared using a high pH LC (*right*, DS-42) and a GELFrEE (*left*, DS-43) fractionation approach. Shown are the truncation site plots. *c,* influence of in-source ion activation: Ratio of the number of proline residues determined at X_N_' and X_C_' position. GELFrEE, gel-eluted liquid fraction entrapment electrophoresis; LC, liquid chromatography.

Strikingly, the biological origin of the sample influenced the proteoform truncation patterns. For example, analyses of proteoform identifications from different human tissues (DS-16-a-e) revealed substantial variability in the number of truncated proteoforms (ranging from 81% in kidney to 98% in spleen, Supplemental Fig. S12), with certain truncation sites showing tissue-specific enrichment (Fig. 4*A*). In lung cells, truncations primarily occurred C terminal to lysine, valine, and arginine, while in spleen cells, the preferential truncation was C terminal to valine, and in the small intestine, it was C terminal to arginine and tyrosine. Another example is DS-30-a and DS-30-b, in which two different brain regions of the zebrafish were analyzed (Supplemental Fig. S12). Both the percentage of N and C terminally truncated proteoforms (80% *versus* 60%), as well as the truncation site patterns, differed slightly. Furthermore, DS-37-a analyzed *E. coli* cells cultivated in LB medium and DS-37-b in minimal medium supplemented with acetate as a carbon source (Supplemental Fig. S12). The associated truncation site plots showed similar patterns; however, a considerably higher number of N and C terminally truncated proteoforms were observed for the *E. coli* samples grown with M9 medium (21% *versus* 7%). Moreover, particularly the truncation sites between lysine and arginine (*i.e.*, the lysine-lysine, lysine-arginine, arginine-lysine, and arginine-arginine peptide bonds) were much more prominent in this sample.

Besides the biological differences, sample preparation (including cell lysis, enrichment of proteoforms suitable for TDP, and proteoform separation) had a considerable influence on the truncation patterns (Supplemental Fig. S13). In agreement with previous studies, different cell lysis solutions yielded varying numbers of truncated proteoforms; for example, urea-based buffers were associated with an increased number of truncations (Supplemental Fig. S13, *A* and *B*) (32, 74, 99). Several hypotheses have been presented to explain this observation, including the generation of artificial proteoforms (32, 74) and the enhanced extraction of hydrophilic and membrane proteins (99). Additionally, proteases that remain active during cell lysis can lead to truncated proteoforms (35).

Moreover, various enrichment and fractionation strategies introduced distinct truncation patterns. For example, in the study by Winkels *et al*. (2021) (69), the *E. coli* proteome was fractionated using an offline high pH reversed-phase (DS-42) and a gel-eluted liquid fraction entrapment electrophoresis (DS-43) separation scheme. Although the same sample was used, the two approaches resulted in diverse truncation site patterns, with a bias toward truncations C terminal to arginine, lysine, tyrosine, phenylalanine, leucine, and alanine in the high pH approach and C terminal to aspartate residues in the gel-eluted liquid fraction entrapment electrophoresis workflow (Fig. 4*B*). Other examples are presented in Supplemental Figure S13, demonstrating that different sample preparation approaches of a given sample result in specific truncation sites. In contrast, similar sample preparations yielded similar truncation site frequencies.

In some cases, studies with similar methodological approaches could be grouped: gel-based approaches (*e.g.*, DS-05, DS-14-c, DS-37, DS-43, DS-46) and solid-phase extraction (*e.g.*, DS-14-d, DS-27, DS-50) resulted in the identification of a high number of proteoforms truncated C terminal to aspartate residues (Supplemental Fig. S14). In gel-based workflows, proteoforms are typically reduced prior to electrophoretic separation, for example, using DTT or β-mercaptoethanol, in combination with elevated temperatures. In contrast, solid-phase extraction protocols typically employ acidic solutions. As described above, both elevated temperatures and acidic conditions can result in artificial protein truncations.

Furthermore, online separation prior to MS analysis can introduce artificial truncations of labile peptide bonds (33). Thus, we compared datasets of studies that utilized LC as well as capillary electrophoresis (CE) separation prior to the mass spectrometric analysis (Supplemental Fig. S15). If the proteoform separation influences the occurrence of truncated proteoforms, it would be expected that the truncation site plots would differ between the two separation techniques. DS-03-a and DS-03-b provided proteoform identifications obtained after CE-MS and LC-MS analysis, respectively. Moreover, we reanalyzed the raw files of DS-16 by dividing them into CE- (DS-16-f/g) and LC-based (DS-16-h/i) measurements. In DS-16, CE-MS yielded a higher number of truncated proteoforms than reversed-phase LC-MS; however, only minimal differences were observed in the normalized frequencies of the truncation sites (Supplemental Fig. S15). Moreover, the CE-MS and LC-MS measurements of DS-03 exhibited a similar number of truncated proteoforms and almost identical truncation sites.

Gas-phase separation using FAIMS has been demonstrated to yield a significant increase in TDP proteoform identifications (47, 51). Furthermore, FAIMS can result in artificial fragmentation of glycosylation during glycopeptide analysis (in-FAIMS fragmentation) (100). To investigate whether FAIMS can artificially introduce proteoform truncations, we analyzed the truncated proteoforms reported by two independent TDP studies (DS-10-a/b and DS-14-a/b), which performed TDP experiments with and without FAIMS. In both studies, the application of FAIMS resulted in similar absolute numbers of full-length proteoforms compared to measurements without FAIMS (Supplemental Fig. S16*A*). However, with FAIMS, a substantial increase in truncated proteoforms was observed, with the highest effect on N and C terminally truncated ones (546 compared to 335 [DS-10] and 406 compared to 105 [DS-14], respectively). Normalized truncation site frequencies showed only minor differences, indicating that FAIMS does not substantially introduce artificial truncations. (Supplemental Fig. S16*B*). These observations can be potentially explained by the increased sensitivity when utilizing gas-phase separation with FAIMS, enabling the identification of low-abundant (truncated) proteoforms. Furthermore, the CVs used favored the identification of small proteoforms, additionally resulting in a higher percentage of truncated proteoforms (47, 51).

In-source ion activation is commonly used in TDP to minimize the formation of noncovalent adducts. However, this process can artificially truncate proteoforms through a CID-like mechanism, leading to the generation of b- and y-ions (Supplemental Fig. S17*A*) (34). Notably, only y-ions share the same masses as truncated proteoforms resulting from peptide bond hydrolysis in solution and, thus, can be easily identified with commonly used database search engines. Additionally, peptide bonds N terminal to proline residues are particularly susceptible to CID-like processes (101). Therefore, we hypothesized that in-source fragmentation should result in the identification of a high number of N terminally truncated proteoforms (*i.e.*, y-ions), particularly affecting peptide bonds N terminal (X_N_' position) to proline residues, whereas these biases are not expected for the proteoform C terminus (X_C_' position). We validated this hypothesis by performing LC-MS/MS experiments of an *E. coli* lysate with different in-source ion activation energies (0–80 V). After proteoform identification with ProSightPD, we determined the number of truncated proteoforms and the frequency of proline residues at the X_N_' and X_C_' positions (Supplemental Fig. S17, *B* and *C*). Above an in-source ion activation energy of 30 V, the number of identified proteoforms truncated at the N terminus gradually increased. In contrast, the number of proteoforms truncated at the C terminus stayed at the same level. Moreover, the ratio of X_N_'/X_C_' for proline residues increased with the in-source ion activation energy, validating our hypothesis.

Thus, we calculated the ratio of proline residues in X_N_' and X_C_' positions for all datasets (Fig. 4*C*). Among all datasets, only DS-15 exhibited a markedly elevated ratio. Furthermore, the truncation site plots of the proteoform termini revealed a distinct bias of proline at the X_N_′ position, particularly between the proline-valine peptide bond (34). In contrast, no such bias was observed at the X_C_′ position (Supplemental Fig. S17, *D* and *E*). These findings suggest that, apart from DS-15, in-source fragmentation contributes minimally to the overall landscape of truncated proteoforms. As the in-source ion activation energy is not provided for DS-15, no final conclusions can be drawn regarding the use of unusually high values. Note that the occurrence of in-source fragmented proteoforms can theoretically also be studied by analyzing coeluting mass features (Supplemental Fig. S17*C*) (34). However, many datasets do not provide elution time information or lack accessible raw data. Thus, examining the ratio of proline residues in the X_N_' and X_C_' positions can serve as a widely applicable alternative for determining in-source fragmentation events.

Finally, we evaluated the influence of the database search on truncated proteoform identification by exemplarily re-analyzing the raw files of DS-14 using ProSightPD and TopPIC (Supplemental Fig. S18). Moreover, different database search settings were applied for ProSightPD, that is, different proteoform databases (based on a FASTA file or an XML file containing all modifications deposited in UniProt), precursor tolerances (10 ppm or 2.2 Da for the full-length proteoform search), and filter criteria (without applying a C-score filter or using a threshold >40). Both the type of database search engines and the various search settings resulted in different numbers of proteoform identifications (Supplemental Fig. S18*A*). In general, more proteoforms were identified if a larger proteoform database, a wider precursor tolerance, and lower quality criteria were utilized. Moreover, all of the mentioned criteria substantially influenced the identification of full-length and truncated proteoforms. For example, performing a database search with ProSightPD using an XML file, a wide precursor tolerance, and no C-score filter resulted in the identification of 21% (n = 754) full-length proteoforms, whereas using a FASTA file and a narrow precursor tolerance resulted in only 8% (n = 250) full-length proteoforms. The analysis with TopPIC yielded the highest number of identifications, with 16% full-length proteoforms. Interestingly, a much lower percentage (17%) of N and C terminally truncated proteoforms was identified than the analysis with ProSightPD (20–31%, depending on the search setting) (83, 85). Notably, despite the database search influencing the number of truncated proteoforms, the truncation sites were remarkably similar (Supplemental Fig. S18*B*), indicating that no bias toward specific truncation sites was introduced.

### Consistent Identification of Truncations Across Various Datasets

We next analyzed whether independent TDP studies consistently reported the same truncation sites in given proteins. To visualize the identified proteoform termini, we plotted their positions relative to the canonical sequence of the protein (hereinafter referred to as termini plots). By arranging the various studies in a stack, consistently identified termini can be easily detected. Termini plots of various proteins are shown in Supplemental Figure S19. For example, the cytochrome b-c1 complex (P47985, human) has been consistently identified with a noncanonical C terminus at position 78 and a noncanonical N terminus at position 79 in nine and eight TDP studies, respectively (Supplemental Fig. S19*C*). This truncation aligns with information in the UniProt database: The N-terminal proteoform (1–78) corresponds to the Cytochrome b-c1 complex subunit 9, while the C-terminal proteoform (79–274) corresponds to the mitochondrial Rieske subunit.

Termini plots were generated for all proteins reported in the datasets, and the number of consistent truncations was counted (Supplemental Fig. S20). For human datasets, the vast majority of truncated proteoform N termini were identified in a single dataset only, that is, they were not reported by other studies (Supplemental Fig. S20*A*). Generally, only a low absolute number of truncations was consistently identified across a high number of datasets; for example, only 30 truncated N termini have been reported by 10 or more independent TDP studies. On the other hand, however, a number of truncations were consistently identified in more than three datasets.

Interestingly, the termini consistently reported in independent TDP studies were, in many cases, also described in UniProt, supporting the hypothesis that the consistent identification of truncated proteoforms across multiple independent TDP studies provides strong hints at biological relevance, that is, that these truncated proteoforms may fulfill important molecular functions. In general, the findings for the C-terminal proteoform mirror those of the N terminus (Supplemental Fig. S20*B*); however, up to now, considerably fewer noncanonical termini have been listed in UniProt. For example, a total of 24 noncanonical C termini have been reported in at least eight independent TDP studies, with only 8 (33%) documented in UniProt. Similar results were observed in the *E. coli* and mice datasets (Supplemental Fig. S20, *C*–*F*).

**Fig. 5:**
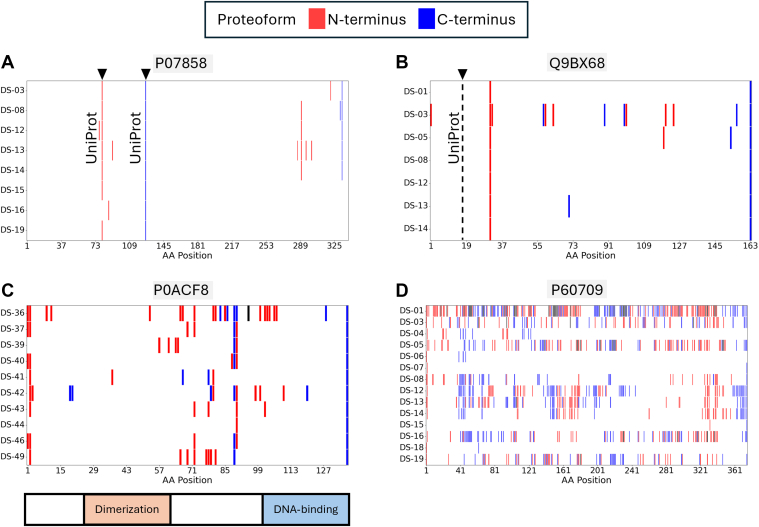
**Overview of neo-termini consistently identified in multiple independent TDP studies.** Represented are the reported proteoform N termini (*red*) and C termini (*blue*) relative to the canonical sequence. The *black triangles* and the *dashed lines* indicate the truncation sites that are deposited in the UniProt Knowledgebase. *A*, the cathepsin B light chain (position 80–126), which is a proteolytic product of the protein cathepsin B (P07858, human), was consistently identified in various TDP studies. *B*, the transit peptide of adenosine 5′-monophosphoramidase HINT2 (Q9BX68, human) is located 13 amino acids N-terminal from the identified N terminus in seven independent TDP studies. *C*, a truncation of the DNA-binding protein H-NS (P0ACF8, *Escherichia coli*) results in two proteoforms, with one covering the dimerization and the other the DNA-binding domain. *D*, the highly abundant protein actin (P60709, human) was identified with multiple termini across the entire encoded protein sequence. TDP, top-down proteomics.

The termini plots can be grouped into several distinct categories, reflecting different types of truncated proteoforms, including termini (i) that correspond to annotated truncation sites in UniProt (Fig. 5*A*); (ii) near or in addition to truncations described in UniProt (Fig. 5*B*); (iii) located between annotated protein domains (Fig. 5*C*); and (iv) with no apparent relation to known or predicted truncation sites (Fig. 5*D*).

For example, in seven TDP studies, the adenosine 5′-monophosphoramidase HINT2 (Q9BX68, human) was identified with a truncated N terminus at residue 31, whereas the expected N terminus at position 18 (corresponding to the annotated signal peptide cleavage site in UniProt) was not detected (Fig. 5*B*). Furthermore, the BolA-like protein 3 (Q53S33, human) was identified in nine studies with an N terminus at residue 27. While no truncation is annotated in UniProt, TargetP (88) predicts a signal peptide with a cleavage site between residues 26 and 27 (Supplemental Fig. S21).

Notably, many truncation events between protein domains were consistently identified across multiple TDP studies. For example, the DNA-binding protein H-NS (P0ACF8, *E. coli*) has an N-terminal dimerization domain (position 21–63) and a C-terminal DNA-binding domain (position 92–137). Nine TDP studies identified a truncation between amino acid positions 89 and 90, resulting in two proteoforms: one encompassing the dimerization domain and another DNA-binding domain (Fig. 5*C*). Furthermore, the LIM and SH3 domain protein 1 (Q14847, human) has consistently been identified with a proteoform that exclusively covers the C-terminal SH3 domain. Other examples include the truncation of SH3 or UBA domains (Supplemental Fig. S22). Besides that, some proteins were identified with proteoforms spanning the transit or propeptide (Supplemental Fig. S23, *A* and *B*), potentially mispredicted translation initiation sites (Supplemental Fig. S23*C*), or with ladder-like termini (Supplemental Fig. S24).

## Discussion

In this meta-analysis, 50 TDP datasets published over the past decade were evaluated to investigate the occurrence and characteristics of truncated proteoforms. Approximately 71% of the reported proteoforms were truncated, with the majority of these truncated species not being documented in UniProt up to now. This high frequency of truncation events exceeds numbers estimated from BUP-based terminomics studies, which have reported that more than 50% of proteins have at least one noncanonical N terminus (3).

The high proportion of truncated proteoforms, compared to the full-length ones, reported in the TDP studies raises the question of (i) whether they originate from biological processes, such as the usage of alternative translation initiation sites or proteolytic processing or (ii) whether they are a product of artifacts introduced at some stage of the analytical pipeline. While the present meta-study cannot answer this question, inferences can still be made regarding the origin of a truncation event. Here, we highlight the critical parameters and factors for future methodological improvements toward enabling TDP to decipher biologically relevant processes.

The consistent identification of certain proteoform termini across independent TDP studies strongly indicates their biological relevance. This hypothesis is supported by the fact that many truncated proteoforms repeatedly identified have already been deposited in UniProt (such as the removal of signal or propeptides and the maturation of proteoforms) (2), and, therefore, have been identified or verified by other methodologies and can be considered biologically relevant.

Our PAPT revealed missannotations in protein databases, such as incorrect signal peptide cleavage sites, and may provide evidence for alternative or secondary cleavage events, thereby supporting the refinement and curation of protein sequence databases. In several cases, N-terminal truncations have been consistently identified in independent TDP studies, deviating from those reported in UniProt (31, 61), or could be attributed to previously unknown signal peptides, as further validated by bioinformatic prediction tools. Additionally, we found several truncated proteoforms in which proteolytic cleavage occurred between structured protein domains (11), that is, events that likely alter protein localization, interaction networks, enzymatic activity, and overall biological function (10). Thus, TDP enables the identification of proteoforms with potentially novel functions, underscoring that proteolysis is not merely a mechanism of protein degradation but a fundamental regulator of protein homeostasis and a key contributor to physiological and pathophysiological processes.

Further, truncated proteoforms identified in different sample types (such as various tissues or cultivation conditions) exhibited distinct truncation site patterns, suggesting a biological origin. This finding aligns with previous TDP studies in which multiple truncated proteoforms of a given protein were identified with opposite differential abundance under varying conditions, indicating distinct biological functions (16, 57, 66, 102).

The second potential source contributing to the high number of truncated proteoforms observed in the studies is the introduction of artifacts during the analytical workflow or an inherent bias associated with the TDP approach itself. TDP is a relatively young discipline that still faces numerous challenges throughout the entire analytical pipeline, for example, the efficient separation of proteoforms and intact protein MS, especially for larger proteoforms (103). These factors lead to a reduced sensitivity and a significant bias toward the identification of proteoforms smaller than approximately 30 kDa (104). The correlation between proteoform size and truncation state, as observed in our meta-analysis, therefore, inherently translates into a high percentage of truncated proteoforms. Generally, the abundance (inferred from the number of PrSMs) of the truncated proteoforms was reduced compared to that of the full-length ones, which is also in agreement with previous reports (9, 31). Consequently, study designs that increase overall sensitivity, such as utilizing multidimensional liquid-based or online gas-phase–based fractionation schemes, also show a clear trend toward the identification of an increased number of truncated proteoforms.

On the other hand, truncated proteoforms can originate from artifacts during the sample preparation, separation, or MS analysis (32). Key factors influencing the occurrence of artifacts identified in this meta-analysis are elevated temperatures (*e.g.*, during reduction of disulfide bridges) (32) and acidic conditions (*e.g.*, during online proteoform separation prior to MS analysis) (33), which can induce peptide bond hydrolysis, particularly of the chemically labile aspartate-proline bond (31, 94). As both BUP and TDP share several common steps in the analytical pipeline, these artifacts will also likely occur in peptide-centric analysis. In BUP, however, peptides derived from artificially truncated proteoforms are still inferred to be the same protein as those from full-length proteoforms. Thus, artificial truncations typically do not impact protein identification and often will remain unnoticed in classical BUP-based analyses. Besides this, in-source fragmentation and gas-phase fractionation with FAIMS only contributed minimally to the generation of artificially truncated proteoforms. The negligible influence of in-source fragmentation is consistent with a previous BUP study investigating in-source fragmentation of tryptic peptides, where it contributed only about 1 to 3% to the total peptides identified (34). In our analysis, where we tested various in-source ion activation energies, significant in-source fragmentation was only observed above a voltage of approximately 30 V. However, typically, the in-source ion activation energy utilized in TDP studies in Orbitrap-based instruments is around 15 V.

The database search affects the number and relative abundance of truncated proteoforms but does not introduce any bias regarding the truncation sites. A higher precursor tolerance likely has a greater impact on the identification of full-length (*i.e.*, longer) proteoforms due to their higher modification capacity. Moreover, the combination of expanded proteoform databases and wide precursor tolerances influences the proportion of full-length proteoforms. Another important factor in the identification of truncated proteoforms may be the control of false discovery rates. Identifying truncated proteoforms requires search algorithms that operate within a large search space (105), which can result in a higher percentage of false-positive identifications. A significant challenge arises from the multitude of possible precursor masses. Since accurate deconvolution is crucial, any errors in this process, in combination with a high number of possible precursor masses, can lead to incorrect identifications (106). This highlights the necessity for implementing additional quality controls to ensure the reliable identification of (truncated) proteoforms.

Based on the results presented in this meta-analysis, a set of recommendations for future studies targeting the analytics of truncated proteoforms can be created: (i) Quality control of the truncated proteoforms, for example, by visualizing the truncation sites using truncation site plots, allowing for an efficient assessment of whether specific truncation sites appear disproportionately frequently, which can help identify their potential origin. Integrating the visualization directly into database search pipelines or visualization tools, such as FLASHApp (107), could further simplify the reproducible analysis of truncated proteoforms. (ii) Elevated temperatures and prolonged incubation under acidic conditions should be avoided. (iii) To further increase confidence in the identified truncated proteoforms, complementary sample preparation methods or proteomics approaches (such as terminal labeling or BUP-based terminomics) (20, 31) should be considered. (iv) The consistent identification of certain proteoform termini across various independent studies strongly suggests biological relevance. Thus, comparing studies on the same organism can help identify biologically relevant truncated proteoforms.

However, there are a number of open questions that require further methodological development at all stages of the analytical pipeline. An urgent issue is how to distinguish between biologically derived and artificially introduced truncated proteoforms. In this regard, terminal labeling approaches shortly after or during cell lysis (or even in living cells) may be a possibility to pinpoint biologically derived proteoforms that are not formed during the sample preparation. While the usability of intact protein N-terminal labeling via reductive demethylation has been demonstrated (31), C-terminal labeling strategies are more challenging, and to date, no protocol for TDP has been presented.

Furthermore, identifying and reducing the sources of possible artifacts is a main task that will also be helpful for BUP-based approaches. One major bottleneck in this respect is the lack of universally accepted standards for reporting proteoform identifications or experimental details. While these standards are well elaborated in BUP (108), there is a need to adapt the guidelines for proteoform-based TDP analyses. For example, several TDP studies do not provide access to raw data or proteoform identification lists, and crucial experimental (such as all reagents used in the experiment or specific durations and temperatures utilized for all steps in the workflow) and identification details (such as PTMs, proteoform retention times, quality scores, and number of PrSMs) are not reported. However, the reusability of both proteoform identifications and raw data is essential to ensure FAIR (109) data practices in TDP (110), enabling further in-depth analyses, for example, to examine the crosstalk between truncations and other PTMs, and contributing to comprehensive resources such as proteoform atlases (111). Furthermore, standardized reporting of experimental details would allow for more straightforward comparisons between datasets. Such comparisons could reveal systematic differences or similarities in experimental workflows, which in turn may help explain recurring patterns in truncation sites across different datasets. A further challenge is elucidating the cause of the low consistency of truncated proteoforms, as a considerable number of truncations were uniquely identified in single studies. These truncations may be specific to certain samples (29), or they might result from exopeptidase activity or protein degradation.

Moreover, a key limitation of TDP is its inability to determine whether truncations occur at the genomic, transcriptomic, or proteomic level. Dissecting these regulatory layers of proteoform generation requires complementary workflows, such as proteogenomics approaches, including long-read sequencing (112). Furthermore, the identification of modified termini can provide further insights (*e.g.*, acetylated truncated N-termini may indicate the presence of an alternative starting site initiation) (9); however, this requires more powerful database search engines (31).

In summary, the comprehensive meta-analysis performed here highlighted multiple factors influencing the occurrence of truncated proteoforms, encompassing both biologically derived variants and artifacts introduced throughout the TDP workflow. Moreover, we showed that the consistent identification of proteoform termini across independent studies indicates biological relevance. Our data highlight the utility of building a comprehensive proteoform atlas to enable the elucidation of consistently identified proteoforms. A concise and structured overview at the individual proteoform level would greatly enhance interpretability and facilitate biological insight. The data generated here provide a resource for investigating truncated proteoforms from specific proteins (Supplemental Table S4) and can serve as a valuable starting point for future molecular and biochemical investigations. Integrating TDP data and other proteomics (*e.g.*, terminomics (3) and “integrative multilevel proteoformics” (113)) or transcriptomics (4) data will further help to unravel the complexity of the proteome and its layers of regulation. This study underscores the significant potential of TDP despite ongoing challenges in this emerging field. With numerous protocols established, substantial methodological advancements are expected in the coming years, enhancing TDP's capabilities and applications.

## Data Availability

All raw data acquired in this study have been uploaded to the ProteomeXchange Consortium (76) via the PRIDE partner repository with the dataset identifier PXD066719. Information related to the proteoform identifications extracted from the literature data is presented in the Supplemental Data.

## Supplemental Data

This article contains supplemental data: Supplemental Figures S1–S24 and Supplemental Tables S1–S4.

## Conflict of Interest

The authors declare no competing interests.
