# Supplementary material for: Properties, Origin, and Consistency of Truncated Proteoforms Across Top-Down Proteomic Studies
Source: Mol Cell Proteomics. 2025 Nov 12;24(12):101465. doi: 10.1016/j.mcpro.2025.101465 (PMC12753228; doi:10.1016/j.mcpro.2025.101465)
Supplement: Supplementary Information [file mmc1.docx]

*Supporting Information*

Properties, Origin, and Consistency of Truncated Proteoforms Across Top-Down Proteomic Studies

Philipp T. Kaulich^1^, James M. Fulcher^2^, and Andreas Tholey^1^*

^1^Systematic Proteome Research & Bioanalytics, Institute for Experimental Medicine, Christian-Albrechts-Universität zu Kiel, Kiel, Germany

^2^Environmental Molecular Sciences Laboratory, Pacific Northwest National Laboratory, Richland, WA, USA

* to whom correspondence should be addressed:

Andreas Tholey

Systematic Proteome Research & Bioanalytics, Institute for Experimental Medicine

Christian-Albrechts-Universität zu Kiel

24105 Kiel, Germany

Phone: #49 (431) 50030300; Fax: #49 (431) 50030308

E-mail: a.tholey@iem.uni-kiel.de

# Table of Contents

[Table of Contents 2](#_Toc212529943)

[1 Supplementary Figures 3](#_Toc212529944)

[2 Supplementary Tables 25](#_Toc212529945)

[3 References 27](#_Toc212529946)

# Supplementary Figures


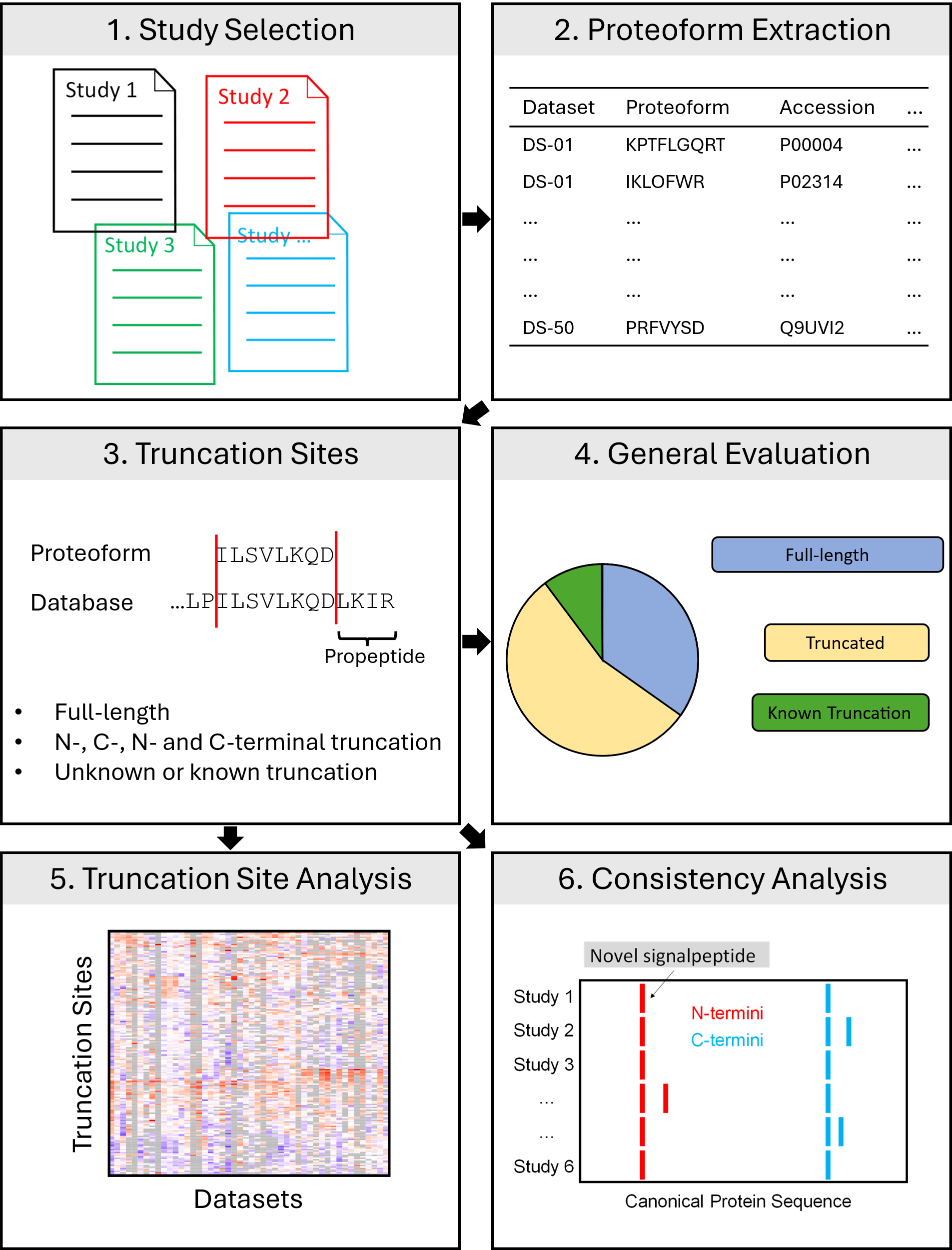


**Supplementary Figure S1: Overview of the Pooled Analysis of Protein Truncation (PAPT)**. 1) Selection of suitable TDP studies that provide proteoform identification lists in their supplementary information or data repositories. 2) Extraction of proteoform sequences and corresponding protein accessions. 3) Determination of the truncation state of each proteoform. 4) General evaluation, including the proportion of truncated proteoforms and the fraction of known (i.e., UniProt-documented) truncations. 5) Truncation site analysis by calculating truncation site frequencies relative to the occurrence of each peptide bond. 6) Consistency analysis and visualization of truncation sites of given proteins across all studies to elucidate novel and likely biological relevant truncation sites.

**Supplementary Figure S2: Number of proteins, proteoforms, and truncation events analyzed from the selected datasets**. Each proteoform truncated at either the N-terminus or the C-terminus is considered as one truncation event. If truncation occurs at both termini, it is counted as two distinct truncation events.

**Supplementary Figure S3: Number of truncated and full-length proteoforms identified in the individual datasets**. **a** Relative number of truncated and full-length proteoforms. **b** Relative number of proteins identified exclusively with truncated, full-length, or truncated and full-length proteoforms.

**Supplementary Figure S4: Influence of encoded protein size on the number of truncation sites and influence of identified proteoform length on the truncation state**. **a** Correlation of the encoded protein length with the number of determined truncation sites. **b** Relationship of proteoform length and truncation events.

**Supplementary Figure S5: Abundance of truncated and full-length proteoforms**. Proteoform plots with the color-coded number of PrSMs as an approximation for their abundance for the proteins **a** P18669 (DS-13), **b** P09211 (DS-05), **c** P30086 (DS-08), and **d** P04908 (DS-13).

**Supplementary Figure S6: Relative position of the truncation sites near the canonical protein termini**. **a,b** Positions of the N-terminal truncation events of (**a**) all datasets and (**b**) selected datasets (the canonical N-terminus is considered as position 1, bin size: 1). The frequently occurring truncation positions are labeled and typically can be explained by the truncation of signal- or transit peptides. **c,d** Positions of the C-terminal truncation events of (**c**) all datasets and (**d**) selected datasets (the canonical C-terminus is considered as position 0, bin size: 1).

**Supplementary Figure S7: Knowledge about the truncated proteoforms**. **a** Percentage of the identified truncated proteoforms that are documented in the UniProt database (release 2024_01)^1^. **b** Average number of proteoform spectrum matches (PrSMs) assigned to truncated proteoforms that have (red) or have not (grey) been deposited in the UniProt database.

**Supplementary Figure S8: Truncation site plots**. The truncated proteoforms were matched to the full-length sequence, and the amino acids N- and C-terminal of the truncation site(s) were denoted as X and X', respectively. Truncation site plots of **a** DS-01, **b** DS-03, **c** DS-13, and **d** DS-08 are displayed.

**Supplementary Figure S9: Amino acids involved in truncation sites and differences of the truncation sites between the proteoform N- and C-termini**. **a** Relative frequency of amino acids involved in truncation sites, which correlates with their relative abundance in the proteome. **b** N-bias of the amino acids across all datasets. The N-bias (adapted from Tabb et al., 2003)^2^ is defined as the logarithmic ratio of the amino acid frequency at position X′ and position X. A positive value indicates that the amino acid is preferentially truncated at the N-terminus and *vice versa*. Proline showed the highest N-bias across nearly all datasets, which aligns with its known structural features that favor hydrolysis of the peptide bond N-terminal to proline residues. **c** The median normalized frequencies of the truncation sites of the proteoform N- and C-termini are shown. The truncation sites that are substantially more frequently observed at the proteoform N- and C-termini are labeled in blue and orange, respectively. The red dotted line represents the line of equality. Truncation sites that are more frequently observed at the proteoform N-terminus are, for example, between alanine-proline, methionine-proline, and methionine-serine residues. This observation could potentially be explained by the fact that the alanine-proline peptide bond is often involved in truncations of signal peptides localized at the proteoform N-terminus. Moreover, the overrepresented truncations between the methionine-serine and methionine-proline residues at the proteoform N-terminus provide hints for the usage of alternative translation initiation sites, with a subsequent excision of the methionine.

**Supplementary Figure S10**: **Truncation site plots with characteristic patterns**. **a,b** The high frequency of truncations N- and C-terminal to alanine residues (e.g., in (**a**) DS-36 and (**b**) DS-32) can be attributed to the cleavage of signal or transit peptides. **c** The truncations C-terminal to proline residues (DS-02) mainly originate from histone proteins with the canonical N-terminal sequence Met-Pro, where the non-canonical proteoform N-terminus is located at position 3 relative to the encoded canonical protein sequence. **d** The characteristic truncations between Ala-Phe, Arg-Gly, and Ser-Arg in DS-23 can be explained by the fact that islet cells were analyzed, resulting in the identification of many proteoforms of the highly abundant protein insulin (P01325, P01326, mouse): The signal peptide of insulin is cleaved at Ala-Phe, the B-chain at Ser-Arg, and the A-chain at Arg-Gly.

**Supplementary Figure S11: Intra-study reproducibility regarding the number of truncated proteoforms**. **a** Number of reported full-length and truncated proteoforms. **b** Normalized frequencies of the truncation sites. DS-28 performed three independent measurements of single *Caenorhabditis elegans* nematodes; DS-40 analyzed three replicates of two different zebrafish brain regions (labeled as Cb and Teo); DS34 performed a long-term reproducibility study of capillary electrophoresis by injecting the same sample multiple times (displayed are the proteoforms reported for the replicates 1, 10, 20, 30, 40, 50, and 62); and DS-46 performed two replicates of two different gel-based sample preparation protocols.

**Supplementary Figure S12: Influence of the sample origin on the identification of truncated proteoforms**. **a** Number and percentage of full-length and truncated proteoforms identified in different human tissues (DS-16), *Escherichia coli* cultivated under different media (DS-37), and two zebrafish brain regions (DS-30). **b** Normalized frequencies of the truncation sites.

**Supplementary Figure S13: Influence of the sample preparation on the identification of truncated proteoforms**. **a** Number and percentage of full-length and truncated proteoforms. **b** Normalized truncation site frequencies. **c,d,e** Truncation site plots of truncated proteoforms identified in DS-14 after sample preparation with (**c**) gel-eluted liquid fraction entrapment electrophoresis (GELFrEE), (**d**) solid-phase extraction (SPE), and (**e**) molecular weight cut-off filter (MWCO).

**Supplementary Figure S14: Certain sample preparation protocols resulted in similar truncation sites.** Truncation site plots of datasets from studies utilizing **a** gel-based (DS-05, DS-14-c, DS-37, DS-43, DS-46) or **b** solid-phase extraction-based (DS-14-d, DS-27, DS-50) sample preparation, showing a bias toward the truncation between aspartate-proline residues.

**Supplementary Figure S15: Influence of the proteoform separation mode on the identification of truncated proteoforms**. **a** Number and percentage of full-length and truncated proteoforms identified in DS-16 and DS-03 after online proteoform separation using capillary electrophoresis (CE) or liquid chromatography (LC) prior to MS analysis. **b** Normalized frequencies of the truncation sites.

**Supplementary Figure S16: Influence of the gas-phase fractionation technique (high-field asymmetric waveform ion mobility spectrometry, FAIMS) on the identification of truncated proteoforms**. **a** Number of full-length and truncated proteoforms with and without (w/o) FAIMS in DS-10 and DS-14. **b** Normalized frequencies of the truncation sites.

**Supplementary Figure S17: Influence of in-source ion activation on the identification of truncated proteoforms**. **a** In-source fragmentation results in the generation of b- and y-ions. Note that the generated y-ions have the same mass as a genuine proteoform (i.e., formed in-solution due to peptide bond hydrolysis), while the b-ions have a mass that is reduced by one oxygen and one hydrogen atom (i.e., −17 Da). As a result, a database search cannot differentiate between actual proteoforms and y-ions that are artifacts of in-source fragmentation. **b,c** Experimental validation performing LC-MS measurements with *Escherichia coli* lysate utilizing in-source fragmentation voltage from 0 V to 80 V. In (**b**), the ratio of the number of proline residues determined at the X_N_' and X_C_' position multiplied by the number of proline residues in X_N_’ position, and in (**c**), the number of co-eluting MS1 mass features is shown. **d,e** Truncation site plots of the proteoform (**d**) N-termini and (**e**) C-termini from DS-15.

**Supplementary Figure S18: Influence of the database search engine and settings on the identification of truncated proteoforms**. The raw files of DS-14 were reanalyzed using the database search engines ProSightPD (PS) and TopPIC and different database search settings: Proteoform database based on a FASTA or XML file, wide window acquisition (WW, 2.2 Da vs. 10 ppm), C-score filter (CS, no filter vs. >40). **a** Number and percentage of full-length and truncated proteoforms. **b** Normalized truncation site frequencies.


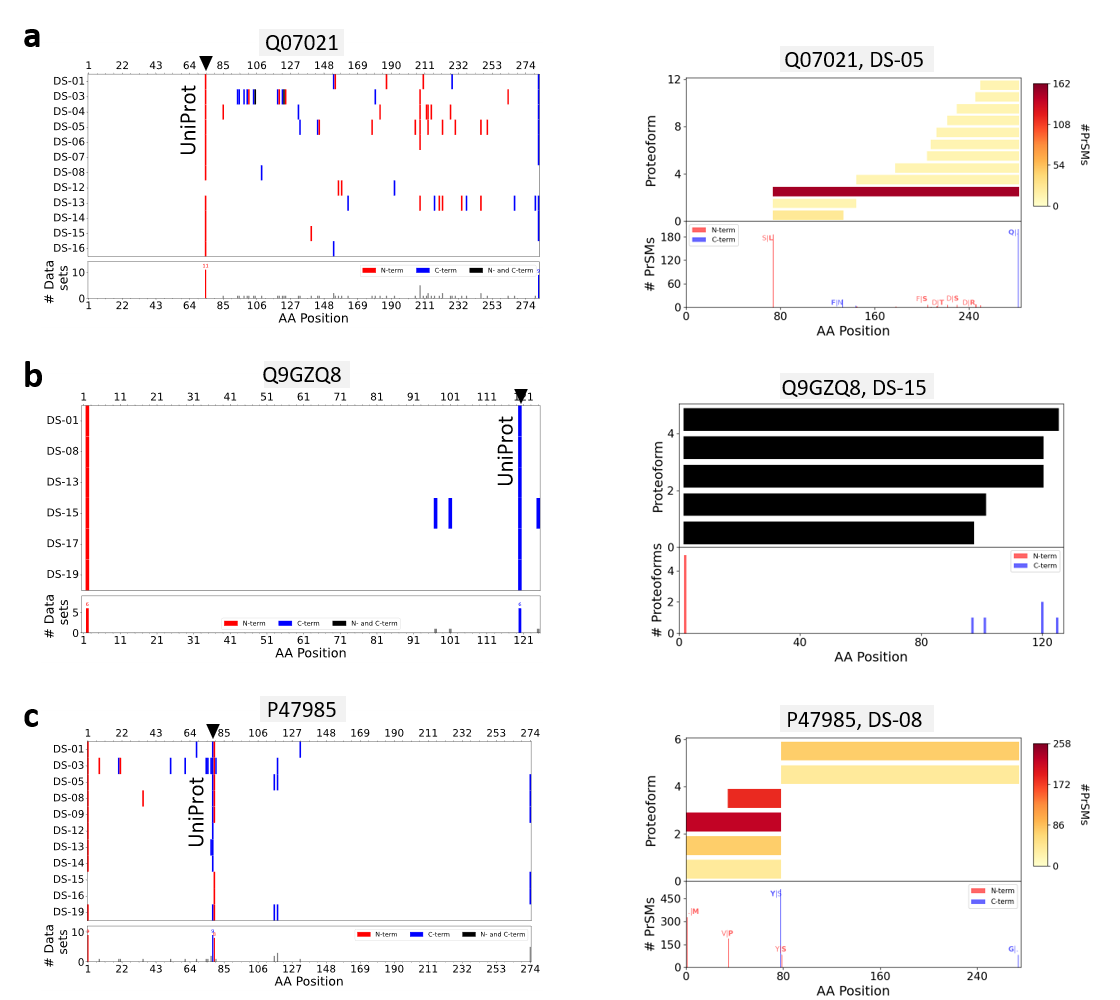


**Supplementary Figure S19: Consistently identified truncation sites that are documented in the UniProt database**. **a** Cleaved signal peptide (position 1-73) of the mitochondrial complement component 1 Q subcomponent-binding protein (Q07021, human). **b** Cleaved propeptide (position 121-125) of the microtubule-associated protein (Q9GZQ8, human). **c** The mitochondrial cytochrome b-c1 complex subunit Rieske (P47985, human) is proteolytically processed between positions 78 and 79, resulting in two independent proteoforms, the subunit 9 (position 1-78) and subunit Rieske (position 89-274). Shown are the termini plots (left panels; representing the identified proteoform N- (red) and C-termini (blue) relative to the canonical sequence) and the proteoform plots from selected datasets (right panels; displaying the reported proteoform sequences with, if available, the color code representing the number of PrSMs per proteoform).

**Supplementary Figure S20: Consistently identified truncation sites**. The relative numbers of truncations consistently identified across different datasets are shown. Truncations deposited in UniProt are indicated in red (described), while those not deposited (undescribed) are shown in grey. The absolute numbers are labeled within each bar. Displayed are **a,b** human, **c,d** *Escherichia coli*, or **e,f** mouse samples.


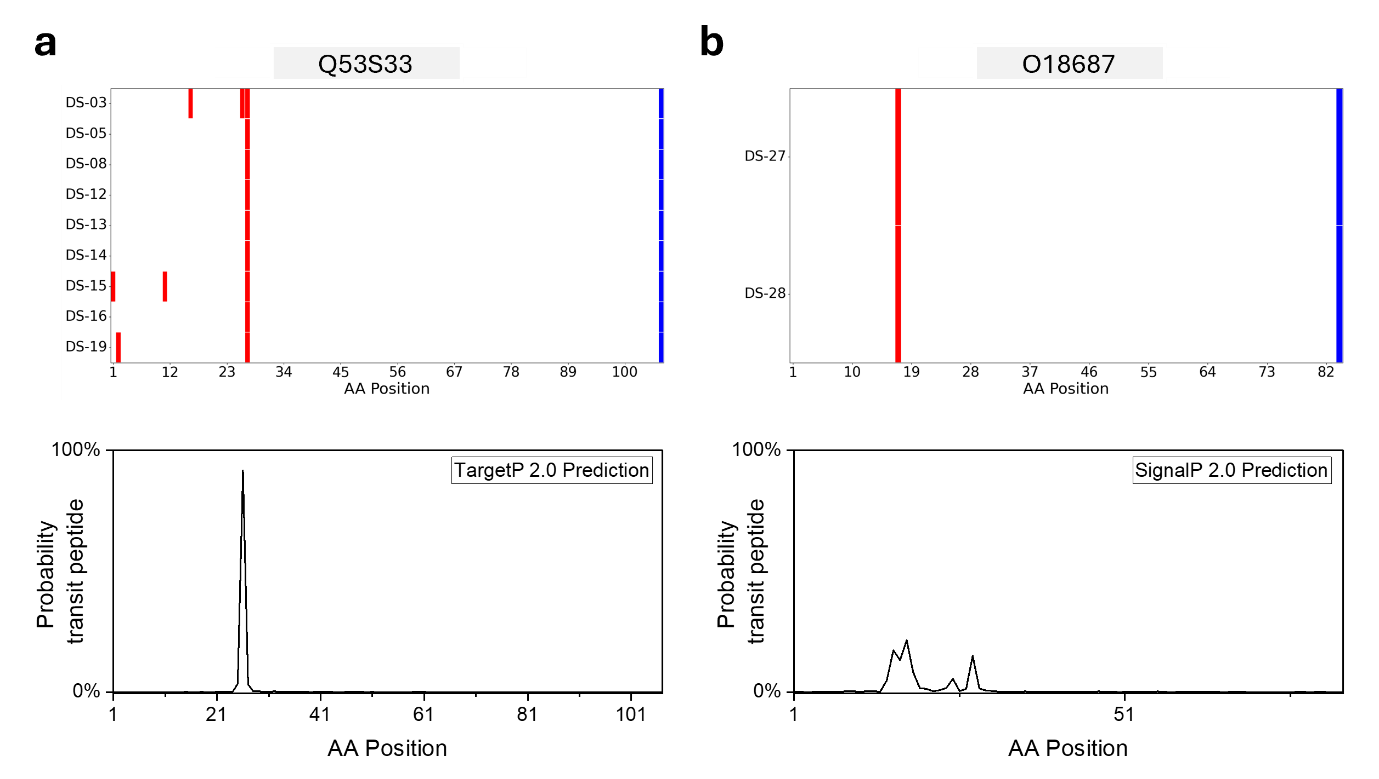


**Supplementary Figure S21: Identification and validation of previously unknown signal peptides**. Shown are the identified proteoform termini (upper panels; displayed are the proteoform N- (red) and C-termini (blue) relative to the canonical sequence) and the prediction of a transitpeptide truncation site by TargetP 2.0.^3^ **a** BolA-like protein 3 (Q53S33, human). **b** Cytochrome c oxidase polypeptide VIIc (O18687, *Caenorhabditis elegans*) protein from.


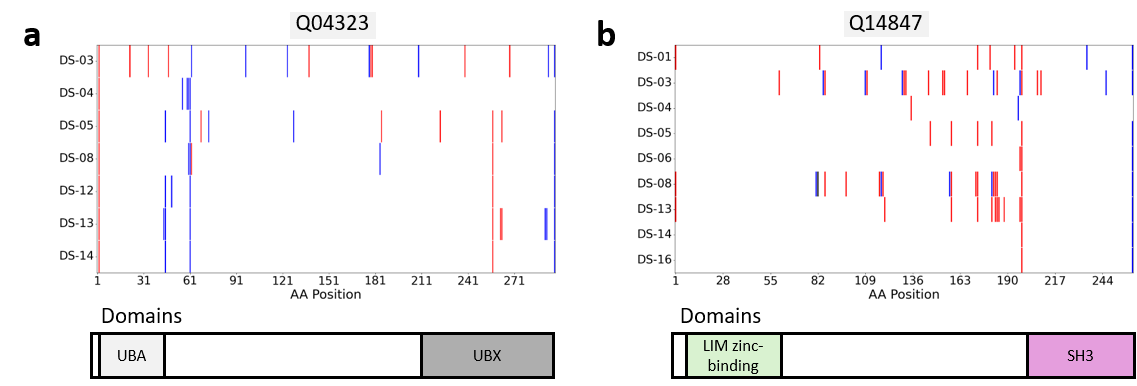


**Supplementary Figure S22: Consistently reported truncation sites between protein domains****.** Displayed are the reported proteoform N- (red) and C-termini (blue) relative to the canonical sequence. **a** The UBX domain-containing protein 1 (Q04323, human) has been consistently identified with two major proteoforms covering the proteoform N- and C-terminus, including the UBA (ubiquitin-associated) and UBX (ubiquitin-regulatory) domain, respectively. **b** The src substrate cortactin protein (Q14247, human) was consistently identified with proteoforms covering the C-terminal SH3 domain.


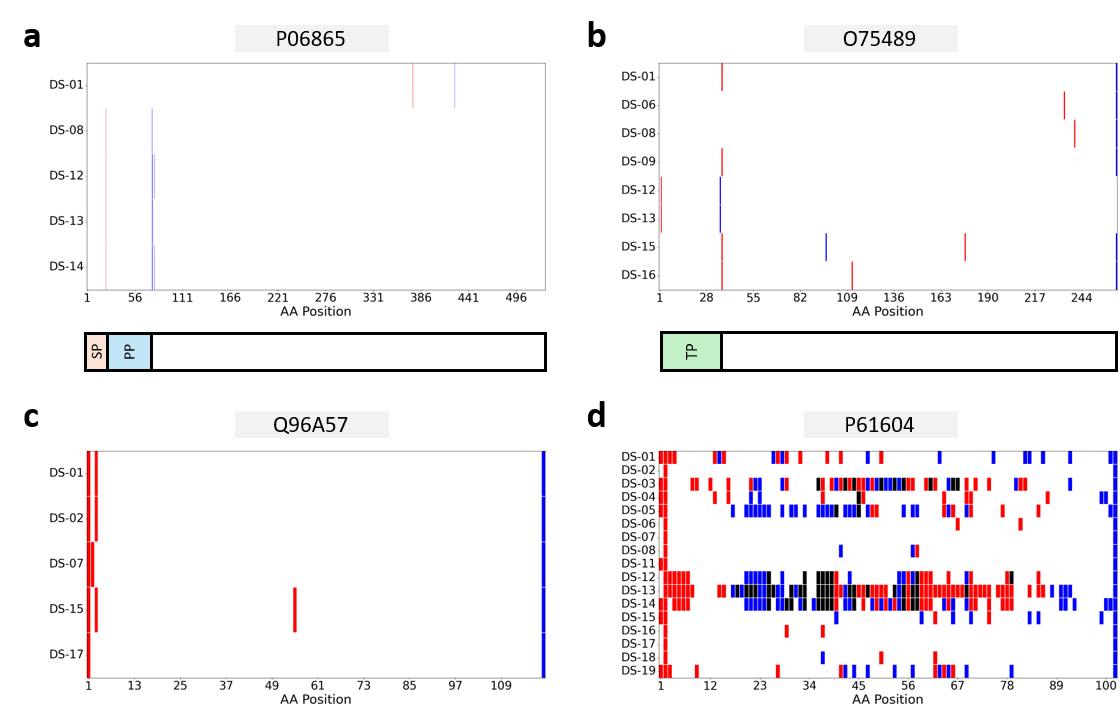


**Supplementary Figure S23: Other observations regarding consistently identified truncations**. Displayed are the reported proteoform N- (red) and C-termini (blue) relative to the canonical sequence. **a**,**b** Identified parts of signal or propeptides. (**a**) The beta-hexosamidinase subunit alpha (P06865, human) is described to have a signal peptide (SP, amino acids 1-22) and a propeptide (PP, 22-88) and was identified in 4 studies with a proteoform covering the amino acids 22-75. (**b**) The mitochondrial NADH dehydrogenase [ubiquinone] iron-sulfur protein 3 (O75489, human) is described to have a transit peptide (TP, position 1-36) and was identified with a proteoform spanning the amino acids 2-36 in two TDP studies. **c** The transmembrane protein 230 (Q96A57, human) was identified in three datasets with a proteoform starting at position 3 (canonical sequence: MMP). **d** Identification of a high number of termini across the entire protein sequence of the 10 kDa heat shock protein (P61604, human).

**Supplementary Figure S24: Ladder-like truncation events**. Proteoform plots of the proteoforms identified in different studies from the **a,b,c** Histone H2B type 1-O (P23527, human) and **d,e,f** large ribosomal subunit protein P2 (P05387, human), highlighting the occurrence of ladder-like N- or C-terminal truncation events. If information about proteoform spectrum matches (PrSMs) was provided, the color code represents the number of PrSMs per proteoform.

# Supplementary Tables

**Supplementary Table S1**: Overview of the datasets utilized for analyzing the influence of various factors on the identification of truncated proteoforms.

| **DS** | **Comment** | **Nomenclature** | **Description** |
| --- | --- | --- | --- |
| DS-03 | LC- or CE-MS analysis | a | CE-MS |
|  |  | b | LC-MS |
| DS-10 | Measurements with and w/o FAIMS | a | Analysis without utilizing FAIMS |
|  |  | b | Analysis utilizing FAIMS |
| DS-14 | Measurements with and w/o FAIMS | a | Analysis without utilizing FAIMS |
|  |  | b | Analysis utilizing FAIMS |
|  | Different sample preparation | c | GELFrEE |
|  |  | d | Solid-phase extraction |
|  |  | e | Molecular weight cut-off filter |
| DS-16 | Different Human tissues | a | Lung |
|  |  | b | Small Intestinal |
|  |  | c | Spleen |
|  |  | d | Heart |
|  |  | e | Kidney |
|  | LC- or CE-MS analysis | f | CE-MS Small Intestinal* |
|  |  | g | CE-MS Spleen* |
|  |  | h | LC-MS Small Intestinal* |
|  |  | i | LC-MS Spleen* |
| DS-28 | Replicates with independent sample preparations | a | 1st replicate |
|  |  | b | 2nd replicate |
|  |  | c | 3rd replicate |
| DS-30 | Different zebrafish brain regions were analyzed in replicates | a | Cerebellum, 1st replicate |
|  |  | b | Cerebellum, 2nd replicate |
|  |  | c | Cerebellum, 3rd replicate |
|  |  | d | Optic tectum, 1st replicate |
|  |  | e | Optic tectum, 2nd replicate |
|  |  | f | Optic tectum, 3rd replicate |
| DS-34 | Injection of the sample multiple times | a | 1st replicate |
|  |  | b | 10th replicate |
|  |  | c | 20th replicate |
|  |  | d | 30th replicate |
|  |  | e | 40th replicate |
|  |  | f | 50th replicate |
|  |  | g | 62nd replicate |
| DS-37 | Different *Escherichia coli* cultivation conditions | a | LB medium |
|  |  | b | M9 medium |
| DS-46 | Different Gel-based sample preparations in replicates | a | PEPPI, 1st replicate |
|  |  | b | PEPPI, 2nd replicate |
|  |  | c | GELFrEE, 1st replicate |
|  |  | d | GELFrEE, 2nd replicate |

*reanalysis of the raw data performed in this study

**Supplementary Table S2**: Utilized proteoforms in this meta-analysis, reported by the various TDP studies.

**Supplementary Table S3**: Normalized frequencies of truncated peptide bonds relative to their occurrence in the datasets. The number of truncation events between two amino acid residues was divided by the number of corresponding peptide bonds identified in the dataset, and the logarithm was calculated.

**Supplementary Table S4**: Termini of all proteins analyzed in this meta-analysis.

# References

(1) Bateman, A.; Martin, M. J.; Orchard, S.; Magrane, M.; Ahmad, S.; Alpi, E.; Bowler-Barnett, E. H.; Britto, R.; Bye-A-Jee, H.; Cukura, A.; Denny, P.; Dogan, T.; Ebenezer, T. G.; Fan, J.; Garmiri, P.; da Costa Gonzales, L. J.; Hatton-Ellis, E.; Hussein, A.; Ignatchenko, A.; Insana, G.; Ishtiaq, R.; Joshi, V.; Jyothi, D.; Kandasaamy, S.; Lock, A.; Luciani, A.; Lugaric, M.; Luo, J.; Lussi, Y.; MacDougall, A.; Madeira, F.; Mahmoudy, M.; Mishra, A.; Moulang, K.; Nightingale, A.; Pundir, S.; Qi, G.; Raj, S.; Raposo, P.; Rice, D. L.; Saidi, R.; Santos, R.; Speretta, E.; Stephenson, J.; Totoo, P.; Turner, E.; Tyagi, N.; Vasudev, P.; Warner, K.; Watkins, X.; Zaru, R.; Zellner, H.; Bridge, A. J.; Aimo, L.; Argoud-Puy, G.; Auchincloss, A. H.; Axelsen, K. B.; Bansal, P.; Baratin, D.; Batista Neto, T. M.; Blatter, M. C.; Bolleman, J. T.; Boutet, E.; Breuza, L.; Gil, B. C.; Casals-Casas, C.; Echioukh, K. C.; Coudert, E.; Cuche, B.; de Castro, E.; Estreicher, A.; Famiglietti, M. L.; Feuermann, M.; Gasteiger, E.; Gaudet, P.; Gehant, S.; Gerritsen, V.; Gos, A.; Gruaz, N.; Hulo, C.; Hyka-Nouspikel, N.; Jungo, F.; Kerhornou, A.; Le Mercier, P.; Lieberherr, D.; Masson, P.; Morgat, A.; Muthukrishnan, V.; Paesano, S.; Pedruzzi, I.; Pilbout, S.; Pourcel, L.; Poux, S.; Pozzato, M.; Pruess, M.; Redaschi, N.; Rivoire, C.; Sigrist, C. J. A.; Sonesson, K.; Sundaram, S.; Wu, C. H.; Arighi, C. N.; Arminski, L.; Chen, C.; Chen, Y.; Huang, H.; Laiho, K.; McGarvey, P.; Natale, D. A.; Ross, K.; Vinayaka, C. R.; Wang, Q.; Wang, Y.; Zhang, J. UniProt: The Universal Protein Knowledgebase in 2023. *Nucleic Acids Res* **2023**, *51* (D1), D523–D531. https://doi.org/10.1093/nar/gkac1052.

(2) Tabb, D. L.; Smith, L. L.; Breci, L. A.; Wysocki, V. H.; Lin, D.; Yates, J. R. Statistical Characterization of Ion Trap Tandem Mass Spectra from Doubly Charged Tryptic Peptides. *Anal Chem* **2003**, *75* (5), 1155–1163. https://doi.org/10.1021/ac026122m.

(3) Armenteros, J. J. A.; Salvatore, M.; Emanuelsson, O.; Winther, O.; Von Heijne, G.; Elofsson, A.; Nielsen, H. Detecting Sequence Signals in Targeting Peptides Using Deep Learning. *Life Sci Alliance* **2019**, *2* (5), e201900429. https://doi.org/10.26508/lsa.201900429.
